# H4K5 lactylation exacerbates acute myocarditis by driving a positive feedback loop between inflammation and metabolic dysfunction

**DOI:** 10.3389/fimmu.2026.1896701

**Published:** 2026-08-24

**Authors:** Yawei Zhao, Yue Cai, Song Shen, Yuchen Wen, Tianyue Li, Lina Kang, Biao Xu, Xuan Sun

**Affiliations:** Department of Cardiology, Cardiovascular Disease Center, Jiangsu Key Laboratory for Cardiovascular Information and Health Engineering Medicine, Nanjing Drum Tower Hospital, Medical School, Nanjing University, Nanjing, China

**Keywords:** acute myocarditis, H4K5la, histone lactylation, immune metabolism, macrophage

## Abstract

Acute myocarditis is a non-ischemic cardiomyopathy with rapid onset and high mortality. Immunometabolic reprogramming of macrophages is a key pathological feature. However the understanding of its mechanisms remains to be clarified. In this study, we found that acute myocarditis induced an increase in glycolysis and lactate accumulation. Inhibition of lactate production ameliorated myocardial injury. Further, we demonstrated that lactate promoted a pro-inflammatory transition of macrophages, thereby amplifying the inflammatory response. Inhibition of lactate in macrophage shifted its phenotype from a pro-inflammatory to an anti-inflammatory subtype. We also observed a significant increase in H4K5 lactylation in macrophages. Next, we identified that H4K5la drove the transcriptional expression of the Rap1, which in turn upregulated the TNF/NF-κB signaling. This lactate-dependent H4K5la was enriched in inflammatory pathways, forming a positive feedback loop that amplified inflammation. Inhibition of Rap1 reduced cardiac inflammation and broke this loop. These consequently lowered H4K5la levels and delayed ventricular remodeling. Collectively, this study reveals the role of H4K5la in promoting inflammatory cascades in myocarditis, and provides a potential therapeutic target for inflammatory cardiomyopathy.

## Introduction

Acute myocarditis is a well-known complication of infection, which exhibits a broad spectrum of clinical syndromes. It is characterized by overwhelming infiltration of inflammatory cells and non-ischemic myocardial injury, ultimately progressing to chronic heart failure (1). In terms of symptoms, acute myocarditis has a rapid onset, a rapid progression, and a poor prognosis. Additionally, patients may develop inflammatory cardiomyopathy in severe cases (2). Importantly, there is approximately 6–10% of young patients experience sudden cardiac death (3). Therefore, elucidating the mechanisms underlying acute myocarditis indispensable for the prevention and treatment of the subsequent inflammatory cardiomyopathy.

Macrophage is the main effector immune cell involved in myocarditis, exhibiting high functional and phenotypic diversity (4). While cardiac tissue resident macrophage plays an important role in maintaining tissue homeostasis and immune surveillance, myocardial injury triggers cytokine-mediated recruitment of circulating macrophages, exacerbating inflammation. Especially, bone marrow-derived macrophages are recruited by the damage associated molecular patterns (DAMPs) to the local myocardium, and then are activated toward a pro-inflammatory state, producing a large amount of inflammatory cytokines, such as IL-1β, IL-6, and TNF-α. These activated macrophages sustain inflammation through continuous cytokine secretion, forming a feedback loop that drives disease progression (5). Therefore, targeting macrophage mobilization, chemotaxis, and polarization represents a promising therapeutic approach for myocarditis.

Recent studies have demonstrated that macrophage metabolic pattern and metabolites are closely associated with its function (6). During inflammation, macrophages undergo metabolic reprogramming, switching from oxidative phosphorylation to aerobic glycolysis. This shift enables rapid ATP generation and produces glycolytic metabolites such as lactate and reactive oxygen species, which drive robust pro-inflammatory cytokine production. Concurrently, these metabolic changes support bacterial clearance by promoting autophagy, inflammasome activation, and pyroptosis, highlighting the essential role of metabolic reprogramming in macrophage phagocytic activation. However, sustained aerobic glycolysis may negatively impact the immunomonitoring function of macrophages. Furthermore, lactate has been shown to directly enhance the release of inflammatory cytokines from macrophages (7, 8), while inhibition of lactate reduces inflammatory cytokine production (9). Macrophage metabolic reprogramming has become a key research focus in cardiovascular disease treatment (10). While modulating macrophage metabolism is known to influence immune function (11), its specific role and mechanisms in myocarditis require further investigation.

Metabolic intermediates function as signaling molecules that modify proteins and activate pathways controlling energy metabolism and inflammation (12). It is known that lactate uptake and glycolysis levels of cardiac tissues are increased after myocardial injury, which results in the intracellular lactate accumulation (13). Therefore, accumulated lactate regulates gene transcription through lactylation of histone lysine residues, a process that shapes macrophage phenotype and function (14). The effects of histone lactylation depend on the specific site. For instance, lactylation at H3K8 promotes repair and immune balance (15), whereas lactylation at H4K12 drives metabolic disturbances and heightened inflammation in macrophages (16). Although histone lactylation has been implicated in diverse pathological states such as myocardial infarction, Alzheimer’s disease, and immune-mediated disorders (17), its precise modification patterns and functional roles in acute myocarditis remain to be elucidated.

In the present study, we found that acute myocarditis led to the lactate accumulation and subsequent induction of histone lactylation in macrophages, particularly marked elevation of H4K5 lactylation (H4K5la). Using CUT&Tag technology, we demonstrated that H4K5la was significantly enriched at the promoter region of Rap1, promoting its transcription and activating the downstream TNF/NF-κB signaling axis. This cascade further amplified inflammatory cytokine release, ultimately contributing to the development of inflammatory cardiomyopathy. Moreover, H4K5la was associated with immune activation and metabolic dysregulation in macrophages. These findings provide new insights into the immunometabolic mechanisms underlying acute myocarditis and offer potential therapeutic target for inflammatory cardiomyopathy.

## Materials and methods

All experimental animal procedures were conducted in accordance with the guidelines of Directive 2010/63/EU of the European Parliament and were approved by the Institutional Ethics Committee of Nanjing Drum Tower Hospital (Approval No. 2024AE1046). In this study, male wild-type C57BL6/J mice (8 weeks old) obtained from Gem Pharmatech Co. Ltd (Jiangsu, China) were used. The *Ldha* floxed mice (*Ldha*^fl/fl^, Catalog NO.S-CKO-03369) and *Lyz2*^Cre^ mice (Catalog NO.C001358) with a C57BL/6J background were obtained from Cyagen (Suzhou, China). Then the *Ldha*^fl/fl^ mice were crossed with *Lyz2*^Cre^ mice to generate myeloid specific *Ldha* knockout mice (*Ldha*^cko^). All animals were housed under specific pathogen-free conditions at the Nanjing Drum Tower Hospital Animal Facility, with free access to a standard laboratory diet and water. The housing environment was maintained at a constant temperature (25 °C) and humidity (65–70%) under a 12-hour light/dark cycle. Random grouping was employed during the experimental setup. All animal experiments were conducted and analyzed by a blinded researcher. Following the study, mice were humanely euthanized via an intravenous overdose of pentobarbital sodium (200 mg/kg).

The acute myocarditis model was established on mice by intraperitoneal injection of LPS (10 mg/kg, Sigma-Aldrich). Control mice were given with same volume of phosphate-buffered saline (PBS). For pharmacological inhibition of LDHA, mice received intraperitoneal injections of FX-11(2mg/kg, HY-16214, MCE) on the day of LPS administration.

For the induction of experimental autoimmune myocarditis (EAM), the male Balb/c mice (8 weeks old) obtained from Gem Pharmatech Co. Ltd (Jiangsu, China) were used. The Balb/c mice received subcutaneous injections on day 0 and day 7 of 250 μg Myosin H Chain Fragment (Ac-RSLKLMATLFSTYASADR; MCE, HY-P2464) emulsified 1:1 (wt/wt) with complete Freund’s adjuvant (Sigma, F5881) as previously reported (4). In the control group, the mice were injected with complete Freund’s adjuvant. Then the mice were examined with echocardiography and euthanized on day 21 for further examination.

### Echocardiography assessments

Transthoracic echocardiography was performed using a VEVO3100 Biomicroscope (Visual Sonics) to assess left ventricular function after acute myocarditis. M-mode tracings were used to measure left ventricular internal dimensions at end-diastole (LVEDD) and end-systole (LVESD), averaged over at least three consecutive cardiac cycles. Left ventricular ejection fraction (LVEF) and fractional shortening (LVFS) were subsequently calculated. All analyses were conducted by a single observer blinded to the identity of the tracings.

### Histological analysis and immunohistochemistry staining

Left ventricular tissue samples were fixed in 10% neutral-buffered formalin, embedded in paraffin, and cut into 8-μm transverse sections. Hematoxylin and eosin (H&E) staining was performed for general morphological evaluation, while Masson’s trichrome staining was used to assess myocardial fibrosis. To detect the size of myocyte, WGA staining were used to visualize the myocyte borders. Then the cardiac sections were incubated at room temperature with WGA-Alexa Fluor 488 (W11261, Invitrogen) for 15 min after deparaffinisation, rehydration and antigen recovery. Then the slides were stained with DAPI for nuclear staining. For each stained section, 10 randomly selected fields were analyzed. The cross-sectional area was calculated by measuring WGA-enclosed areas under blinded conditions using Image-Pro Plus (Version5.0, Media Cybernetics).

To detect the infiltration of macrophages in the myocardium, the heart paraffin sections were incubated with primary antibodies at a dilution of 1:100 for CD68 (ab283654, Abcam), 1:100 for iNOS (ab283655, Abcam) and 1:100 for CD206 (ab300621, Abcam) after deparaffinisation, rehydration and antigen recovery. Then the sections were incubated with anti-rabbit IgG antibody, followed by staining with DAB substrate solution, and nuclear counterstaining with hematoxylin. All images were captured using a microscope (Olympus, Japan). For each stained section, 10 randomly selected fields were analyzed. The immunoreactive cells for CD68, iNOS and CD206 were quantitatively evaluated for number/view of each heart tissue under blinded conditions using Image-Pro Plus software (version 5.0; Media Cybernetics).

### Immunofluorescence and TUNEL staining

To evaluate the levels of lactylation in the injured myocardium, the tissues were embedded in OCT compound (Tissue-Tek OCT) and serially cut into 5 µm sections. Cryosections were fixed in 4% PFA, and blocked with PBS containing 5% goat serum albumin and were permeabilized with 0.1% Triton-X 100 in the blocking solution. Then the sections were incubated with the primary antibodies at a dilution of 1:100 for CD68 (ab283654, Abcam), 1:100 for Panla (PTM-1425RM, PTM Biolabs) or 1:100 for H4K5la (PTM-11407RM, PTM Biolabs) and 1:500 for cTNT (ab8295, Abcam). Next day, secondary antibodies were added as follows: Alexa Flour^®^ 488 1:500, Alexa Flour^®^ 594 1:500 and Alexa Flour^®^ 647 1:500 for 2h at RT. Nuclei were counterstained with DAPI. To identify the apoptosis in the hearts from mice, TUNEL staining was performed in the cryosections according to the manufacturer’s instructions (A113, Vazyme).Then the nuclei were counter-stained with DAPI. Images were taken using a conventional fluorescence microscope (Olympus, Japan). A total of 10 microscopic fields covering 1 mm^2^ of the myocardial area were photographed, and the positive areas were counted by a blinded investigator.

### L-Lactate content detection

The L-lactate content in the serum and the heart tissues were measured with a lactate detection kit (MAK329, Sigma) according to the manufacturer’s instruction. Briefly, plasma samples and heart tissue extracts were diluted at 1:5 prior to the assay. Then the samples were assayed for the concentration of L-lactate on lactate dehydrogenase catalyzed oxidation of lactate, in which the formed NADH reduces a formazan (MTT) reagent. The intensity of the product color, measured at 565 nm, is proportionate to the lactate concentration in the sample.

### Enzyme-linked immunosorbent assay analysis

EDTA-anticoagulated blood samples were collected and incubated on a rocking platform for 30 min at room temperature. Platelet-poor plasma was subsequently obtained by centrifugation at 12,000 rpm for 20 min at 4 °C. Plasma concentrations of IL-1β (EK201B, Multi-Sciences), IL-6 (EK206, Multi-Sciences) and TNF-α (EK282, Multi-Sciences) were measured using ELISA kits according to the manufacturer’s protocols.

### Bone marrow-derived macrophages isolation and culture

Bone marrow-derived macrophages (BMDMs) were isolated from 4–5 weeks adult C57BL/6j mice following established protocols (18). Briefly, bone marrow cells were flushed from the femurs and tibia of mice, filtered to remove debris, and subjected to red blood cell lysis. After centrifugation, the cell pellet was resuspended and cultured in medium containing recombinant mouse macrophage colony-stimulating factor (M-CSF; 10 ng/mL; 315-02, Gibco) for 7 days to generate mature macrophages for subsequent experiments. To induce inflammation in BMDMs, 10 ng/ml LPS (L2880, Sigma) was added into RPMI 1640 medium containing 1% FBS for 24 hours. In addition, BMDMs were incubated with or without LDHA inhibitor FX-11 (40 μM, HY-16214, MCE) for 24 hours.

### Protein extraction and western blotting

Cardiac tissues or BMDMs were lysed in RIPA buffer (Sigma Aldrich), and total protein concentrations were determined using a BCA assay (Thermo Fisher Scientific). Equal amounts of protein were then resolved by 8–12% SDS-PAGE using Mini Protean precast gels (Bio-Rad Laboratories). Protein homogenates were subjected to analysis using antibodies against PKM2 (4053, CST), HK2 (3582, CST), LDHA (2867, CST), Panla (PTM-1425RM, PTM Biolabs), H4K5la (PTM-11407RM, PTM Biolabs), H4K8la (PTM-1415RM, PTM Biolabs), H4K12la (PTM-1411RM, PTM Biolabs), H4 (A19815,Abclonal), iNOS (ab283655, Abcam), Arg1 (ab259271,Abcam), TNFR1 (3736,CST), T-P65 (8242,CST), P-P65 (3033,CST), TNF-α (11948,CST), Rap1 (A7981, Abclonal), Bcl-2 (ab182858, Abcam), Bax (ab32503, Abcam), H4K5ac (PTM-163, PTM Biolabs), P300 (ab10485, Abcam) and β-actin (HRP-66009, Proteintech). Then, the protein homogenates were incubated with horseradish peroxidase-labeled anti-rabbit IgG. The signals were detected with an ECL system (Amersham) and quantified by scanning densitometry with an ImageJ analysis system.

### Real-time polymerase chain reaction analysis

Total RNA was extracted from cultured cells or cardiac tissues using an RNA isolation kit (R401-01, Vazyme Biotech) following the manufacturer’s protocol. cDNA was synthesized from 1 µg of total RNA using a Prime Script RT Reagent Kit (R233-01, Vazyme Biotech). Real-time PCR was carried out on an Applied Biosystems using Power SYBR Green PCR Master Mix (Q341-02, Vazyme Biotech). The thermal cycling conditions were as follows: 95 °C for 5 min, followed by 40 cycles of 95 °C for 10 s and 60 °C for 30 s. Relative gene expression was quantified using the 2^−ΔΔCt method with β-actin or 18S as the internal control. Sequences for all PCR primers (5’-3’) are presented as follows:

IL-1β: Forward: CCAGCTTCAAATCTCACAGCAG;
Reverse: CTTTGGGTATTGCTTGGGATC;
IL-6: Forward: ACAACGATGATGCACTTGCAGA;
Reverse: GATGAATTGGATGGTCTTGGTC;
TNF-α: Forward: ACGGCATGGATCTCAAAGAC;
Reverse: AGATAGCAAATCGGCTGACG;
PKM2: Forward: CAGAGAAGGTCTTCCTGGCTCA;
Reverse: GCCACATCACTGCCTTCAGCAC;
HK2: Forward: CCCTGTGAAGATGTTGCCCACT; Reverse: CCTTCGCTTGCCATTACGCACG;
LDHA: Forward: ACGCAGACAAGGAGCAGTGGAA;
Reverse: ATGCTCTCAGCCAAGTCTGCCA;
Rap1: Forward: GGAGAATGGAGAGGCAGACAAC;
Reverse: CTGGCTGTGTTTCTGAGTCTTCC;
P300: Forward: GTGATGACCCTTCCCAACCTCA;
Reverse: CTCGTGGTGAAGGACACAGATC;
β-actin: Forward: CATTGCTGACAGGATGCAGAAGG;
Reverse: TGCTGGAAGGTGGACAGTGAGG
18S: Forward: CGGCTACCACATCCAAGGAA; Reverse: GCTGGAATTACCGCGGCT.

### Flow cytometry analysis

For flow cytometry analysis, mice were euthanized by intraperitoneal injection of ketamine (120 mg/kg), xylazine (12 mg/kg), and atropine (0.08 mg/kg). Blood samples were collected, followed by retrograde perfusion of the hearts *in situ* with ice-cold PBS containing 1% FBS to remove circulating blood cells. Then the left ventricles were dissected and minced with 0.5mg/ml tissue dissociation collagenase (Liberase™ TH, Roche) using tissue dissociator (Miltenyi Biotec), followed with filtered and centrifuged at 1500 rpm for 10 minutes at 4 °C to collect the single-cell suspension. For blood monocytes detection, 100ul blood were incubated with anti-CD45-APC-Cy7 (557659, BD Biosciences), anti-FVS-eFlour660 (65-0864-14, eBioscience) and anti-CD11b-PerCP-Cyanine5.5 (45-0112-82, eBioscience) for 45 min at 4 °C in the dark. Then, the blood samples were incubated with red blood cell lysis buffer to remove the red cells. For cardiac macrophages detection, the single-cell suspension were incubated with anti-CD45-APC-Cy7 (557659, BD Biosciences), anti-FVS-eFlour660 (65-0864-14, eBioscience) and anti-F4/80-FITC (123107, Biolgend). Then the cells were incubated with Fixation/Permeabilization buffer (554714, BD Biosciences) to staining the intracellular cytokines. Next, the cells were washed and incubated with anti-iNOS-PE (696805, Biolgend) and anti-CD206-APC (141707, Biolgend) for 45 min at 4 °C in the dark. After incubation, the cells were washed and resuspended in 200 µl of PBS with 1% FBS. Data were acquired on a FACS Calibur flow cytometer (Beckman, USA) and analyzed using FlowJo software (version 10.1, Tree Star, Inc.). For flow cytometry gating, doublets were firstly excluded, and then live cells were analyzed after gating on FVS^-^CD45^+^ populations. The circulating monocytes were identified as CD45^+^CD11b^+^ populations in the blood mice. The cardiac total macrophages, M1 macrophages and M2 macrophages were identified as CD45^+^F4/80^+^, CD45^+^F4/80^+^iNOS^+^, CD45^+^F4/80^+^CD206^+^ populations, respectively, in the cardiac tissues.

To detect the apoptosis, the cells or the cardiac single-cell suspension were incubated with the Annexin V-FITC/PI staining buffer (A211-01, Vazyme Biotech) according to the manufacturer’s protocol. For the evaluation of the inflammatory oxidative stress, the cells or the cardiac single-cell suspension were incubated with the DCFDA-FITC fluorescent probe (287810, Sigma) following the manufacturer’s instructions. Data were acquired on a FACS Calibur flow cytometer (Beckman, USA) and analyzed using FlowJo software (version 10.1, Tree Star, Inc.).

### Seahorse flux analysis

BMDMs were seed at the density of 5 x 10^4^ per well on a Seahorse XF96 cell plate with PBS, LPS or LPS+FX-11 treatment. The utility plates containing the injector ports and probes were filled with calibrant solution (200 μl/well) and placed in a CO2-free incubator at 37 °C overnight. The next day, the culture medium was replaced with the XF assay media (pH7.4; 103575-100, Agilent) supplemented with 1 mM pyruvate (103578-100, Agilent), 10 mM glucose (103577-100, Agilent), 2 mM glutamine (103579-100, Agilent), followed by incubation at 37°C in a non-CO_2_ incubator for 1 hour. The oxygen consumption rate (OCR) was measured after the sequential injection of oligomycin (1 μM), FCCP (0.9μM) and rotenone/antimycin A (0.5 μM). For the extracellular acidification rate (ECAR) assay in a separate plate, 10 mM glucose, 0.5 μM oligomycin, and 100 mM 2-deoxy-glucose (2-DG) were sequentially added. All subsequent steps were performed according to manufacturer’s protocol. Basal OCR and ECAR were then determined following the manufacturer’s instructions, and the resulting data were analyzed using Wave Software (Agilent).

### CUT&Tag assay

CUT&Tag experiment was performed with Novo NGS CUT&Tag 4.0 High-Sensitivity Kit (Novoprotein, China) according to the manufacturer’s instructions as previously reported (19). In brief, BMDMs were collected and fixed on the surface of Concanavalin A-coated magnetic beads. Subsequently, cells were incubated with primary antibodies against H4K5la (PTM-11407RM, PTM Biolabs) overnight at 4 °C. Next day, the goat anti-rabbit IgG antibody was incubated for 1.5 hour room temperature. After washing, the samples were incubated with pA-Tn5 transposase, followed with transposon activation and tagmentation. Then, DNA was isolated, amplified and purified for high-throughput sequencing. The library for sequencing was constructed (CUT&Tag Novogene Kit) and the AMPure beads were used for purification steps. The library preparations were sequenced on Illumina Novaseq platform (Novogene Bioinformatic Technology, China) and paired-end reads were generated.

### RNA sequencing

BMDMs RNA were extracted using Trizol reagent (R401-01, Vazyme). The construction of cDNA library of BMDMs and high-throughput sequencing were completed by Bioprofile Technology as previously reported (20). Briefly, RNA-seq libraries were constructed using the TruSeq RNA sample preparation kit (Illumina, San Diego, CA, USA) and sequenced on a NovaSeq 6000 platform (Illumina) following the manufacturer’s protocols. Raw sequencing data in FASTQ format were generated, and low-quality reads were filtered using Cutadapt (v1.15). Clean reads were then aligned to the reference genome using HISAT2 (v2.0.5). Gene expression levels were quantified by counting the number of reads mapped to each gene using HTSeq (v0.9.1), and fragments per kilo base of transcript per million mapped reads (FPKM) were calculated to normalize expression values. Differential expression analysis between groups was performed using the DESeq (v1.30.0) package with the following thresholds: |log_2_FoldChange| > 1 and adjusted P-value < 0.05. To visualize the expression patterns, bidirectional hierarchical clustering analysis of the differentially expressed genes across all samples was conducted using the R package pheatmap (v1.0.8). Additionally, functional enrichment analyses, including Gene Ontology (GO) and Kyoto Encyclopedia of Genes and Genomes (KEGG) pathway annotations, were carried out against the whole transcriptome background, with a Bonferroni-corrected P-value < 0.05 considered statistically significant.

### ChIP-PCR assays

The ChIP-PCR assays were performed with the Pierce Magnetic ChIP Kit (26157, Thermo Fisher) according to the manufacturer’s instructions. In brief, the 4×10^6^ BMDMs were cross linked using 1% formaldehyde for 10 min at room temperature, and the reaction was quenched with glycine at a final concentration of 125 mM for 5 min. Fixed cells were sequentially added into the membrane extraction buffer and MNase digestion buffer supplemented with protease inhibitors and subjected to sonication for three 20-second pulses at 3 watts power (using Sonicator 3000 Instrument) to obtain chromatin fragments averaging 300 bp. For immunoprecipitation, diluted chromatin was incubated overnight at 4 °C with constant rotation with either normal IgG (negative control) or anti-H4K5la antibody, followed by incubation with ChIP Grade Protein A/G Magnetic Beads for an additional 2 hour at 4 °C with mixing. Collect the beads with a magnetic stand and carefully remove and discard the supernatant. Beads were washed sequentially with IP Elution buffer at 65 °C for 30 minutes with vigorous shaking. The eluted IP samples and the input samples were added with DNA binding buffer and pipetted into a DNA Clean-Up Column to harvest the purified DNA. Next, ChIP-PCR analysis was performed using Power SYBR Green PCR Master Mix on an ABI PRISM 7500 system (Applied Biosystems).

### RNA interference

For Rap1 knockdown in BMDMs *in vitro*, the small-interfering RNA (siRNA) kits were obtained from Vigene Biosciences. Three independent siRNA sequences were initially evaluated and the one sequence with the highest silencing efficiency was selected for subsequent experiments. A scrambled siRNA served as a negative control (NC) group. BMDMs were seeded 1 day prior to transfection of Opti-MEM (Invitrogen) without serum supplemented with jetPRIME transfection reagent (Sartorius). Then the siRNAs were added for 24 hours before collection for further analysis. The RNA interference target sequences for Rap1 were listed as follows:

RAP1-1:sense sequence: 5’-CUUCAUCUCCACGCAGUACAUTT-3’, anti-sense sequence: 3’- TTGAAGUAGAGGUGCGUCAUGUA-5’; RAP1-2: sense sequence: 5’- GCCCUGACUUUGAAAUACAUATT-3’, anti-sense sequence: 3’- TTCGGGACUGAAACUUUAUGUAU-5’; RAP1–3 sense sequence: 5’- UCCACUCUGUUCGUGAGAGAATT-3’, anti-sense sequence: 3’- TTAGGUGAGACAAGCACUCUCUU - 5’. Then the RAP1–1 was selected for the subsequent experiments.

For P300 knockdown in BMDMs *in vitro*, the siRNA sequences of P300 was constructed as previously reported (21): sense sequence: 5’- GGUAUGAUGAACAGUCCAGTT-3’, anti-sense sequence: 3’- GGACUACCCUAUCAAGUAATT- 5’.

### Adeno-associated virus encoding Rap1 delivery

For depletion of Rap1 in the macrophages, an adeno-associated virus (AAV) 9 encoding Rap1 with a CD11b promoter (AAV-Rap1) and the control virus AAV9-CD11b-scramble (AAV-vector) were constructed by Vigene Biosciences (Shandong, China). The RAP1–1 sequence was selected for the interference target as listed above. The AAVs were intravenous administered at a dose of 5 × 10^11^ vg/ml per mouse. After 3 weeks, the mice were given LPS (10 mg/kg) via intraperitoneal injection.

### Statistical analysis

Data normality was assessed using the Shapiro-Wilk test. Homogeneity of variances was evaluated using Levene’s test (for t-tests) or Bartlett’s test (for ANOVA). Data are presented as the mean ± SD. The numbers of each group are described in individual figure legends. Statistical comparisons between two groups were conducted by the unpaired Student t-test; Statistical significance between multiple groups was determined using One-way ANOVA followed by Bonferroni’s multiple comparisons test or Two-way ANOVA followed by Bonferroni’s multiple comparisons test. All statistical analyses were performed with Graph Pad Prism 10, and a P < 0.05 was considered statistically significant.

## Results

### Inhibition of lactate production alleviates damage in acute myocarditis

**Figure 1:**
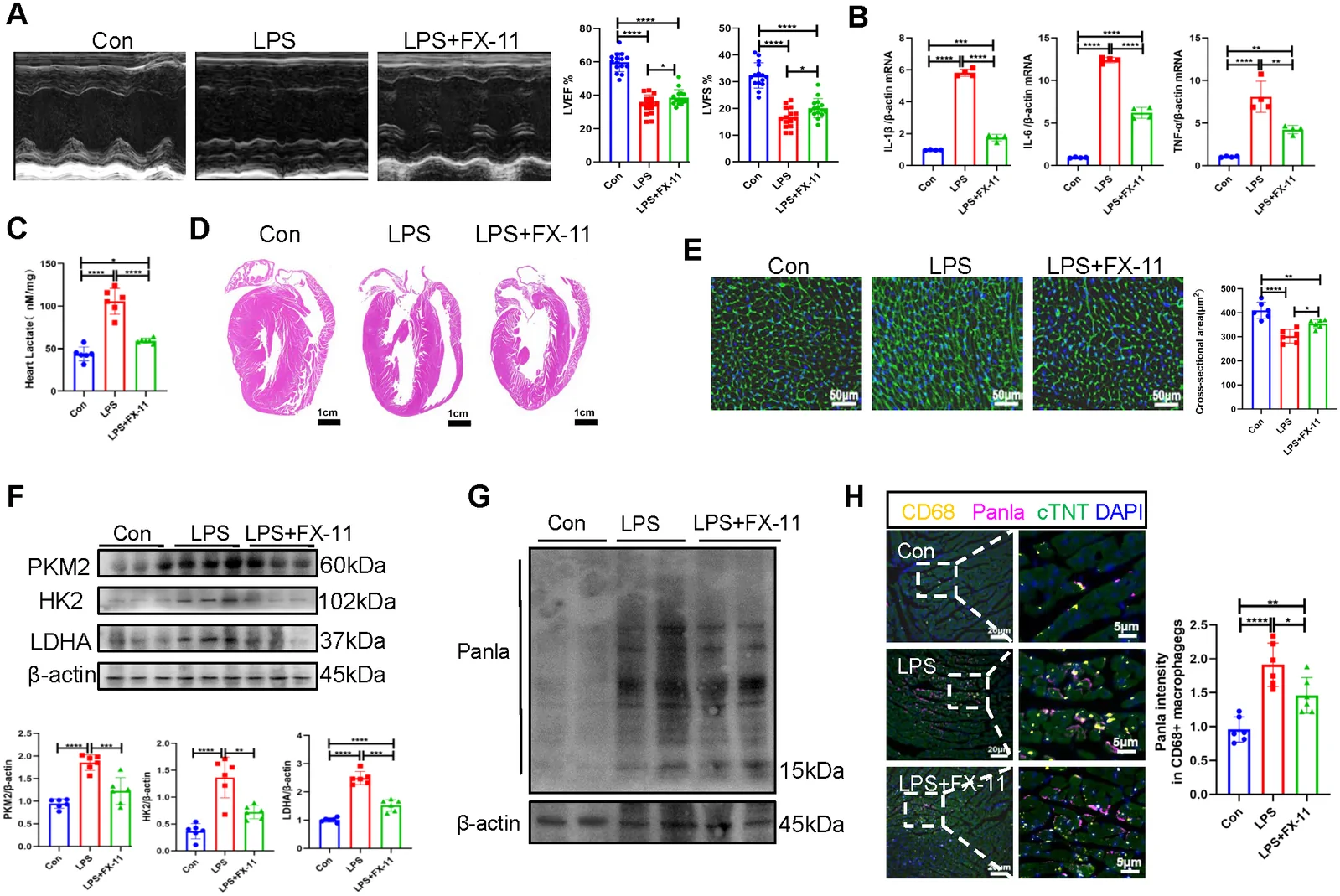
Myocardial damage in acute myocarditis is exacerbated by lactate production. **(A)** Cardiac function was assessed by transthoracic echocardiography at 1 day after acute myocarditis. Left ventricular ejection fraction (LVEF) and left ventricular fraction shortening (LVFS) were calculated in each group (n=15 each group). **(B)** Relative gene expression of IL-1β, IL-6 and TNF-α mRNA was normalized to β-actin mRNA in the cardiac tissues from each group (n=4 each group). **(C)** Quantification of lactate content in the heart tissues (n=6 each group). **(D)** Representative images of HE staining of heart tissues from mice. Scale bar=1 cm. **(E)** Representative immunofluorescence images of cardiac sections stained with myocyte membrane (WGA, green) and nuclei (DAPI, blue) from mice. Scale bar=50 μm. **(F)** Representative immunoblots and the corresponding analysis of glycolysis associated proteins in the cardiac tissues from each group. β-actin was shown as the loading control (n=6 each group). **(G)** Representative immunoblots of Panla protein in the cardiac tissues from each group. β-actin was shown as the loading control. **(H)** Representative immunofluorescence images of cardiac sections stained with macrophage (CD68, yellow), protein lactylation (Panla, pink), myocyte (cTNT, green) and nuclei (DAPI, blue) from mice. Quantifications of Panla^+^CD68^+^ intensity were shown on right (n=6 each group). Scale bar were shown as the indicated in each images. Data were presented as mean ± SD. Data were analyzed by One-way ANOVA followed by Bonferroni test. *p <0.05;**p < 0.01; ***p < 0.005 and ****p < 0.001.

In this study, a murine model of LPS-induced acute myocarditis was established to investigate lactate accumulation during myocardial inflammation. Mice were treated with the lactate dehydrogenase A (LDHA) inhibitor FX-11 following LPS administration to suppress lactate production. Echocardiography assessment revealed that LPS exposure significantly reduced left ventricular ejection fraction (LVEF) and left ventricular fractional shortening (LVFS), indicating impaired cardiac contractile function. Notably, FX-11 treatment attenuated the LPS-induced decline in cardiac function (**Figure 1A**). ELISA analysis showed that inhibiting lactate production significantly reduced the circulating levels of IL-1β, IL-6, and TNF-α (**Supplementary Figure 1A**). Consistently, Q-PCR analysis confirmed the decreased expression of these inflammatory cytokines in cardiac tissue (**Figure 1B**). Assays of lactate contents in both cardiac tissue and serum confirmed that FX-11 treatment markedly reduced lactate production (**Figure 1C**; **Supplementary Figure 1B**). Compared to the LPS group, histological analysis revealed that the thinning of the ventricular wall and the hypertrophy of ventricular myocardial cells were attenuated in the LPS+FX-11 group (**Figures 1D, E**). We also noted that there were more apoptotic myocytes in the LPS group than in the FX-11 treatment group (**Supplementary Figures 1C, D**). In addition, histological analysis at 14 day after myocarditis demonstrated that FX11 significantly ameliorated long-term disturbed cardiac muscle fibers and fibrosis (**Supplementary Figure 1E**). These findings suggest that lactate aggravates myocardial damage and promotes the chronic cardiomyopathy.

Given that lactate production is closely linked to glycolytic flux, we assessed the protein levels of key glycolytic enzymes, including pyruvate kinase M2 (PKM2), hexokinase 2 (HK2), and LDHA, in the cardiac tissues. Immunoblots analysis revealed that the expression of the glycolysis proteins were markedly upregulated in LPS-induced acute myocarditis, an effect that was attenuated by FX-11 treatment (**Figure 1F**). Our results also demonstrated that FX-11 reversed the LPS-mediated elevation in pan-lactylation (Panla) in the hearts from mice (**Figure 1G**). Macrophage plays an important role in the acute myocarditis. Next, we assessed the levels of lactylation in macrophages. Immunofluorescence staining revealed a marked increase in Panla positive macrophages in cardiac tissue following LPS challenge, which was significantly reduced by FX-11 treatment (**Figure 1H**). Immunoblots analysis of LPS-treated bone marrow derived macrophages (BMDMs) showed a substantial elevation in global protein lactylation, with a prominent band at approximately 15 kDa suggestive of the histone modification (**Supplementary Figure 1F**). To further confirm the role of lactate, we employed the α-MyHC-induced experimental autoimmune myocarditis (EAM) model. These results demonstrated that inhibition of lactate production improved cardiac function and attenuated myocardial lactylation (**Supplementary Figures 1G, H**).

Taken together, our findings suggest that lactate accumulation contributes to the acute myocardial injury through the induction of protein lactylation in macrophages within the heart.

### Lactylation accumulation facilitates macrophage polarization

**Figure 2:**
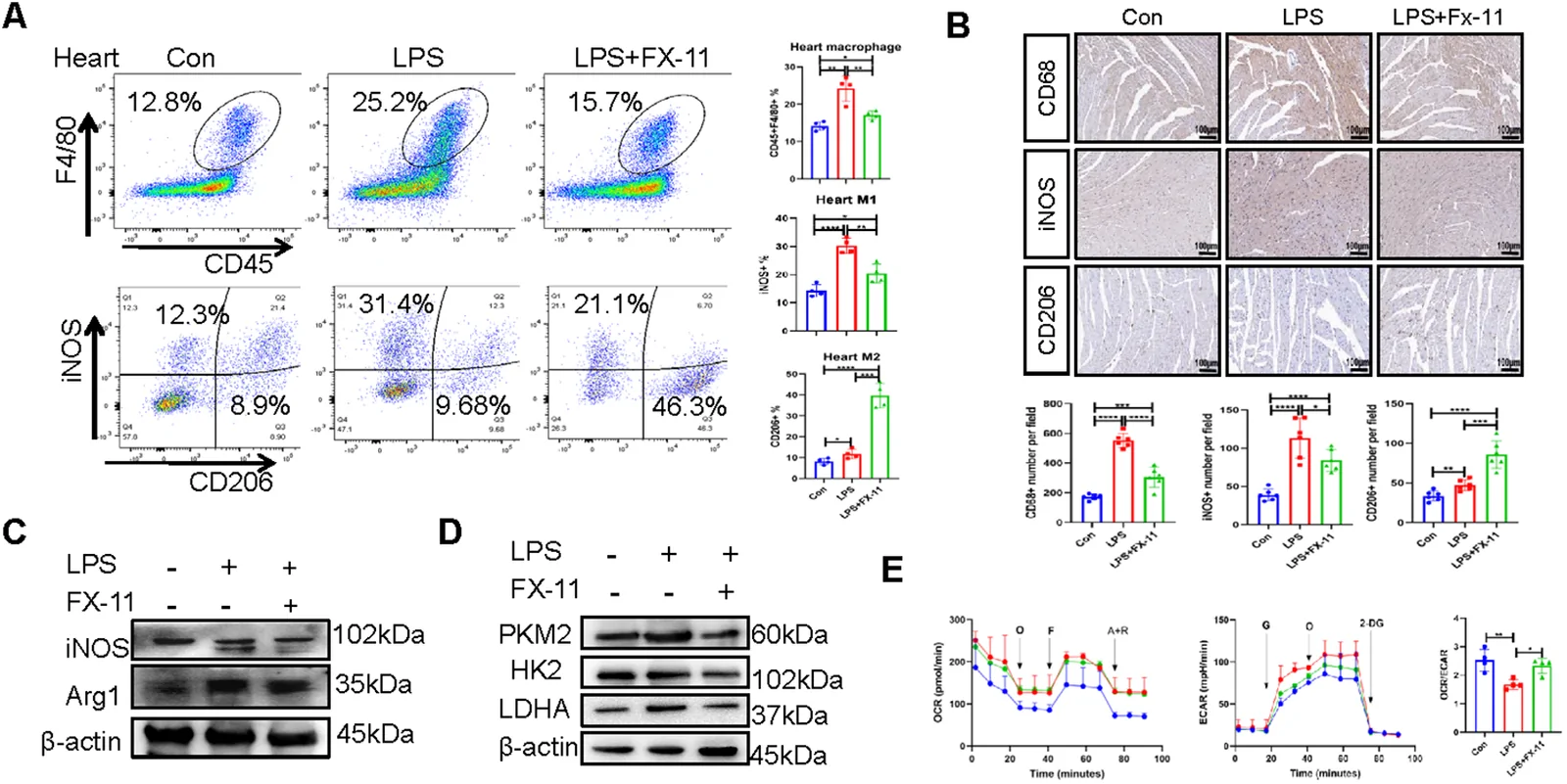
Lactate drives macrophage polarization toward a pro-inflammatory M1 phenotype. **(A)** Representative flow cytometry and statistical analysis of total macrophages, M1 macrophages and M2 macrophages in the cardiac tissues (n=4 each group). **(B)** Representative immunohistochemical images and the corresponding analysis of cardiac tissues staining with macrophage marker CD68, M1 subtype macrophage marker iNOS and M2 subtype macrophage marker CD206 (n=6 each group). Scale bar=100 μm. **(C)** Representative immunoblots and the corresponding analysis of M1 macrophage marker iNOS and M2 macrophage marker Arg1 proteins in the BMDMs treated with LPS and FX-11. β-actin was shown as the loading control. **(D)** Representative immunoblots and the corresponding analysis of glycolysis associated proteins in the BMDMs treated with LPS and FX-11. β-actin was shown as the loading control. **(E)** The oxygen consumption rate (OCR) and the extracellular acidification rate (ECAR) in the BMDMs treated with LPS and FX-11 were detected using a Seahorse XFp analyzer (n=4 biological replicates). For OCR measurement, cells were treated with oligomycin (O), FCCP (F) and antimycin A/rotenone (A+R). For ECAR measurement, cells were treated with glucose (Glc), oligomycin (O), and 2-deoxyglucose (2-DG). Data were presented as mean ± SD. Data were analyzed by One-way ANOVA followed by Bonferroni test. *p <0.05;**p < 0.01; ***p < 0.005 and ****p < 0.001.

To determine whether lactate modulates macrophage responses *in vivo*, we quantified the mobilization, infiltration, and polarization of the macrophages in the circulating and the cardiac tissues. Representative flow cytometry gates for identification of circulating monocytes and cardiac macrophages were depicted in **Figure 2A** and **Supplementary Figures 2A–C**. The results showed that FX-11 significantly inhibited LPS-induced monocyte accumulation in the blood (CD45^+^CD11b^+^) as well as the macrophage infiltration in the myocardium (CD45^+^F4/80^+^) (**Figure 2A**; **Supplementary Figure 2C**). Meanwhile, we observed FX-11 treatment shifted macrophage polarization from a pro-inflammatory M1-subtype toward an anti-inflammatory M2-subtype (**Figure 2A**). Consistently, immunohistochemical staining of heart tissues confirmed that macrophages infiltration and polarization in the myocardium was distinctly altered by FX-11 treatment (**Figure 2B**). Next, the effects of lactate *in vitro* were future verified. BMDMs were treated with different concentrations of lactate and the western blotting showed that lactate modulated macrophage phenotype in a dose-dependent manner, with 10 mM exerting the strongest promoting M1 and inhibiting M2 effects (**Supplementary Figure 2D**). We also observed that FX-11 treatment downregulated M1-subtype marker expression while upregulating M2-subtype markers (**Figure 2C**; **Supplementary Figures 2E**, **S2G**). In addition, inhibiting lactate production protected against macrophage apoptosis and alleviated oxidative stress-mediated inflammatory injury (**Supplementary Figures 2F**, **S2H, I**). It is known that macrophage function is closely associated with its metabolism. The results showed that the glycolysis activity in macrophages increased progressively with rising lactate concentrations (**Supplementary Figure 2J**). Notably, our results demonstrated that FX-11 led to a decline in the expression of the glycolysis proteins when compared to the LPS group (**Figure 2D**). Seahorse analysis further confirmed the metabolic reprogramming in the BMDMs treated with LPS and FX-11 (**Figure 2E**). Therefore, these results revealed that lactate accumulation drove the metabolic shift toward glycolysis in macrophages, subsequently leading to a pro-inflammatory polarization.

### Reduced lactate production in macrophages mitigates myocarditis injury

**Figure 3:**
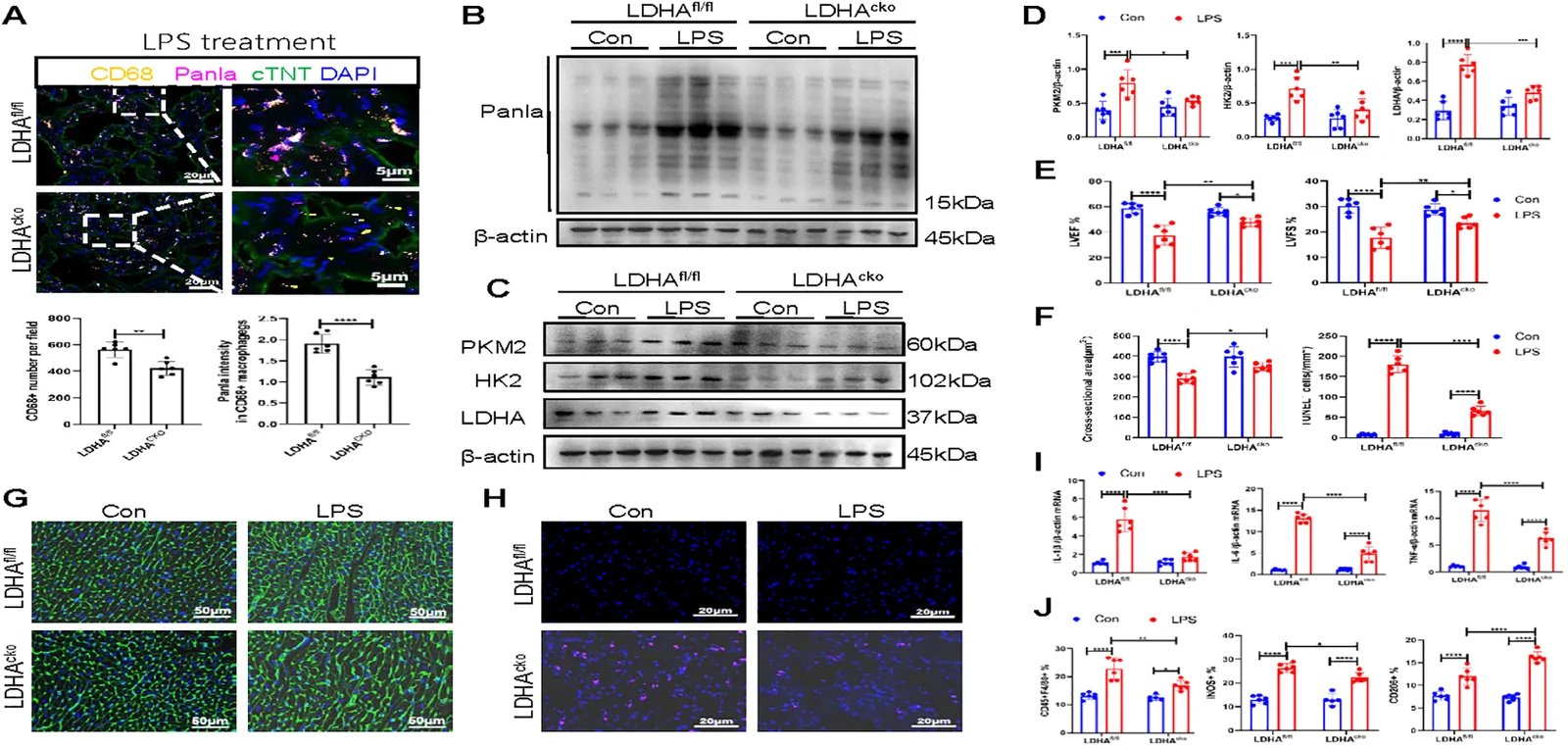
Deficiency in lactate production within macrophages mitigates myocardial injury in myocarditis. **(A)** Representative immunofluorescence images of cardiac sections stained with macrophage (CD68, yellow), protein lactylation (Panla, pink), myocyte (cTNT, green) and nuclei (DAPI, blue) from LDHA^fl/fl^ and LDHA^cko^ mice after LPS treatment. Quantifications of CD68^+^ intensity and Panla^+^CD68^+^ intensity were shown (n=6 each group). Scale bar=50 μm. **(B)** Representative immunoblots of Panla protein in the cardiac tissues from LDHA^fl/fl^ and LDHA^cko^ mice. β-actin was shown as the loading control. C-D. Representative immunoblots and the corresponding analysis of glycolysis associated proteins in the cardiac tissues from LDHA^fl/fl^ and LDHA^cko^ mice. β-actin was shown as the loading control (n=6 each group). **(E)** Echocardiography measurements of cardiac function of LDHA^fl/f^ and LDHA^cko^ mice. LVEF and LVFS were calculated in each group (n=6 each group). **(F–H)** Representative images and the corresponding analysis of WGA **(G)** and TUNEL **(H)** staining of cardiac sections from LDHA^fl/fl^ and LDHA^cko^ mice (n=6 each group). Scale bar were shown as indicated in each images. **(I)** Relative gene expression of IL-1β, IL-6 and TNF-α mRNA was normalized to β-actin mRNA in the cardiac tissues from each group (n=6 each group). **(J)** Flow cytometry analysis of CD45^+^F4/80^+^ total macrophages, iNOS^+^M1 macrophages and CD206^+^M2 macrophages in the cardiac tissues from LDHA^fl/fl^ and LDHA^cko^ mice (n=6 each group). Data were presented as mean ± SD. Data of A were analyzed by student’s t-test; Data of **(D–F, I, J)** were analyzed by Two-way ANOVA followed by Bonferroni test. *p <0.05;**p < 0.01; ***p < 0.005 and ****p < 0.001.

In the view of the central role of macrophage in the acute myocarditis, we wondered the effect of lactate exposure and the macrophages lactylation on acute myocarditis. Considering the majority of cardiac macrophages following LPS treatment were derived from the bone marrow myeloid, we generated the myeloid-specific LDHA knockout mice (LDHA^cko^) by crossing LDHA^fl/fl^ mice with Lyz2^Cre^ transgenic mice. Efficient ablation of LDHA expression was confirmed in BMDMs and the cardiac tissues derived from these mice (**Supplementary Figure 3A**). Subsequently, both LDHA^fl/fl^ and LDHA^cko^ mice were administered LPS. The contents of lactate in the serum and the heart tissues were reduced in the LDHA^cko^ group (**Supplementary Figure 3B**). Immunofluorescence staining revealed that LDHA ablation resulted in a marked decrease in CD68 positive cells as well as Panla positive macrophages in cardiac tissue following LPS treatment (**Figure 3A**). Western blotting showed the lower trend of Panla expression after LDHA knockout (**Figure 3B**). In addition, we observed the reduced expression of glycolysis associated proteins in the LPS-treated LDHA^cko^ mice when compared to the LDHA^fl/fl^ group (**Figures 3C, D**). Next, we assessed the effect of LDHA-specific knockdown on myocardial damage. Echocardiography assessments revealed that myeloid-specific LDHA knockout significantly improved cardiac function in LPS-treated mice, as indicated by preserved LVEF and LVFS (**Figure 3E**). Immunofluorescence analysis of heart sections demonstrated that LDHA deficiency in macrophage significantly inhibited the cardiomyocyte shrinkage and apoptosis (**Figures 3F–H**). Additionally, LDHA^cko^ markedly decreased the levels of pro-inflammatory cytokines, including IL-1β, IL-6 and TNF-α, in both serum and heart tissue (**Figure 3I**; **Supplementary Figure 3C**). Flow cytometry analysis showed that LDHA deficiency inhibited the mobilization of CD45^+^CD11b^+^ monocytes in the circulation as well as the infiltration of CD45^+^F4/80^+^ macrophages in the hearts (**Figure 3J**; **Supplementary Figure 3D**). The results further demonstrated that LDHA^cko^ shifted macrophage polarization from M1 toward M2 phenotype (**Figure 3J**). Moreover, histological examination at 14 day after myocarditis revealed that specific LDHA deletion attenuated LPS-induced cardiac muscle fiber disarray and fibrosis (**Supplementary Figures 3E, F**). Collectively, these data implicate macrophage-derived lactate as a critical mediator of myocardial dysfunction.

### H4K5 lactylation activates transcription of Rap1 in macrophages

**Figure 4:**
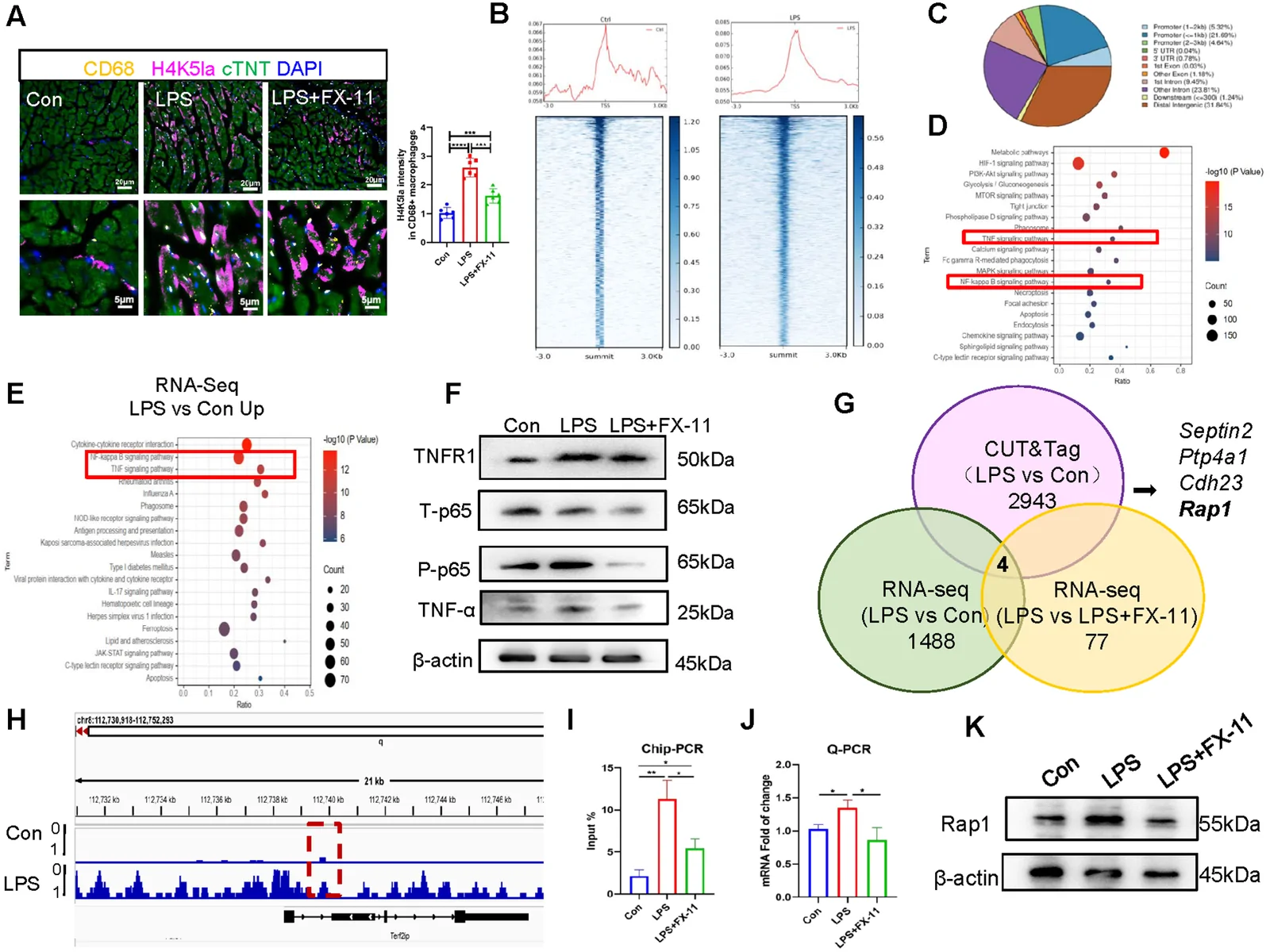
H4K5 lactylation in macrophages directly activates Rap1 transcription via promoter recruitment. **(A)** Representative immunofluorescence images of cardiac sections stained with macrophage (CD68, yellow), H4K5 lactylation (H4K5la, pink), myocyte (cTNT, green) and nuclei (DAPI, blue) from mice. Quantifications of H4K5la^+^CD68^+^ intensity were shown on right (n=6 each group). Scale bar were shown as indicated. **(B)** Heatmaps showed the H4K5la binding density (CUT&Tag tag counts) at individual peaks in LPS-treated macrophages, with peaks sorted by signal strength. **(C)** The genomic landscape of upregulated H4K5la binding peaks was characterized in macrophages. **(D)** The KEGG analysis of the elevated H4K5la binding peaks at candidate target pathway. **(E)** The KEGG enrichment of RNA sequencing in macrophages were presented and ranked. The TNF and NF-κB signaling pathways were both elevated in the CUT&Tag sequencing and RNA sequencing upon LPS activation. **(F)** Representative immunoblots of TNF and NF-kB pathway proteins in the BMDMs treated with LPS and FX-11. β-actin was shown as the loading control. **(G)** The intersection of the up-regulated gene expression in the three datasets. **(H)** The visualization of enrichment of H4K5la at Rap1 loci in the LPS-stimulated BMDMs was shown. **(I)** H4K5la occupancy of Rap1 analysis in BMDMs after LPS and FX-11 treatment by ChIP-PCR. **(J)** Relative gene expression of Rap1 mRNA was normalized to 18S mRNA in the BMDMs treated with LPS and FX-11. **(K)** Representative immunoblots of Rap1 in the BMDMs treated with LPS and FX-11. β-actin was shown as the loading control. Data were presented as mean ± SD. Data were analyzed by One-way ANOVA followed by Bonferroni test. *p <0.05;**p < 0.01; ***p < 0.005 and ****p < 0.001.

Next, we investigated the specific site of lactylation in BMDMs. Previous studies have indicated histone lactylation plays an important role in the macrophage function (14). However, the role of histone H4 lactylation remains unclear. Our results demonstrated that administration of FX-11 significantly reduced H4K5 lactylation in the myocardial tissue (**Figure 4A**; **Supplementary Figure 4A**). *In vitro* experiments showed that lactylation at the H4K5 site in BMDMs increased in a dose-dependent manner with increasing lactate concentrations (**Supplementary Figure 4B**). Similarly, FX-11 treatment decreased LPS-induced H4K5 lactylation in BMDMs (**Supplementary Figure 4C**). Given that H4K5 acetylation has been previously documented (22), we compared the changes in H4K5la and H4K5ac levels under inflammatory stimulation. We observed that H4K5ac levels exhibited an initial increase followed by a decline, whereas H4K5la levels increased progressively with the duration of inflammatory stimulation (**Supplementary Figure 4D**). This indicated that the accumulation of lactylation competitively occupies the H4K5 site, thereby inhibiting acetylation. Therefore, we focused on the H4K5 lactylation for subsequent investigations.

The transcriptional activation or repression of target genes can be regulated by histone modifications. To investigate the gene targets of H4K5la in BMDMs, we performed CUT&Tag assays using an anti-H4K5la antibody. The quality metrics of CUT&Tag assays were shown in the **Supplementary Figure 4E**. Data analysis with deep tools revealed that H4K5la peaks were significantly enriched in LPS-stimulated BMDMs compared to the control group (**Figure 4B**). Moreover, H4K5la was predominantly enriched at promoter regions, with 31.65% of these peaks localized to promoter sequences (≤3 kb) (**Figure 4C**). A total of 2943 up-regulated target genes of H4K5la binding peaks at promoter in LPS-induced BMDMs were classified by gene ontology into different KEGG pathways. As shown in the **Figure 4D**, the KEGG pathway analysis revealed a significant enrichment of inflammation and apoptosis pathways, including the HIF-1, TNF and NF-κB signaling cascades and so on. Subsequently, we performed RNA sequencing on BMDMs stimulated with LPS and treated with FX-11. KEGG pathway enrichment analysis of the transcriptomic data indicated that inflammation and apoptosis pathways, particularly the TNF and NF-κB signaling pathway, were upregulated in the LPS group, highlighting their potential functional relevance (**Figure 4E**). Western blot results confirmed that FX-11 treatment significantly suppressed the LPS-induced activation of TNF and NF-κB signaling, suggesting that inhibition of lactate production alleviates apoptosis and inflammatory damage (**Figure 4F**). Next, differentially expressed genes (DEGs) of RNA-sequencing were analyzed by comparing the LPS group versus the control group and the LPS+FX-11 group versus the LPS group, respectively (**Supplementary Figures 4F, G**). We focused on the genes that were highly expressed in the LPS group but showed low expression in both the control and FX-11-treated groups. RNA-seq heatmap analysis revealed several key genes associated with inflammation and apoptosis, including *Nrg4*, *Cxcr6*, *Terf2ip*, and *Saa3* (**Supplementary Figure 4H**). Integration of CUT&Tag and RNA-sequencing data revealed an overlapping set of genes upregulated by LPS. Among these, we focused on the inflammatory mediator *Terf2ip* (also named Rap1), identifying it as a potential target of lactylation (**Figures 4G, H**). ChIP-PCR analysis confirmed that LPS stimulation markedly enriched H4K5la at the Rap1 promoter in BMDMs, while FX-11 treatment reversed this enrichment (**Figure 4I**). Q-PCR and western blot assays validated the corresponding regulation of Rap1 expression (**Figures 4J, K**). To explore the regulatory mechanism of H4K5la on Rap1, we knocked down the reported writer P300 in macrophages *in vitro* (**Supplementary Figure 4I**). The results demonstrated that P300 knockdown significantly attenuated the LPS-induced increases in H4K5la and Rap1 (**Supplementary Figure 4J**). Collectively, these results suggest that H4K5la promotes Rap1 expression in a p300-dependent manner in macrophages.

### Loss of Rap1 reduces macrophage-mediated inflammatory damage

**Figure 5:**
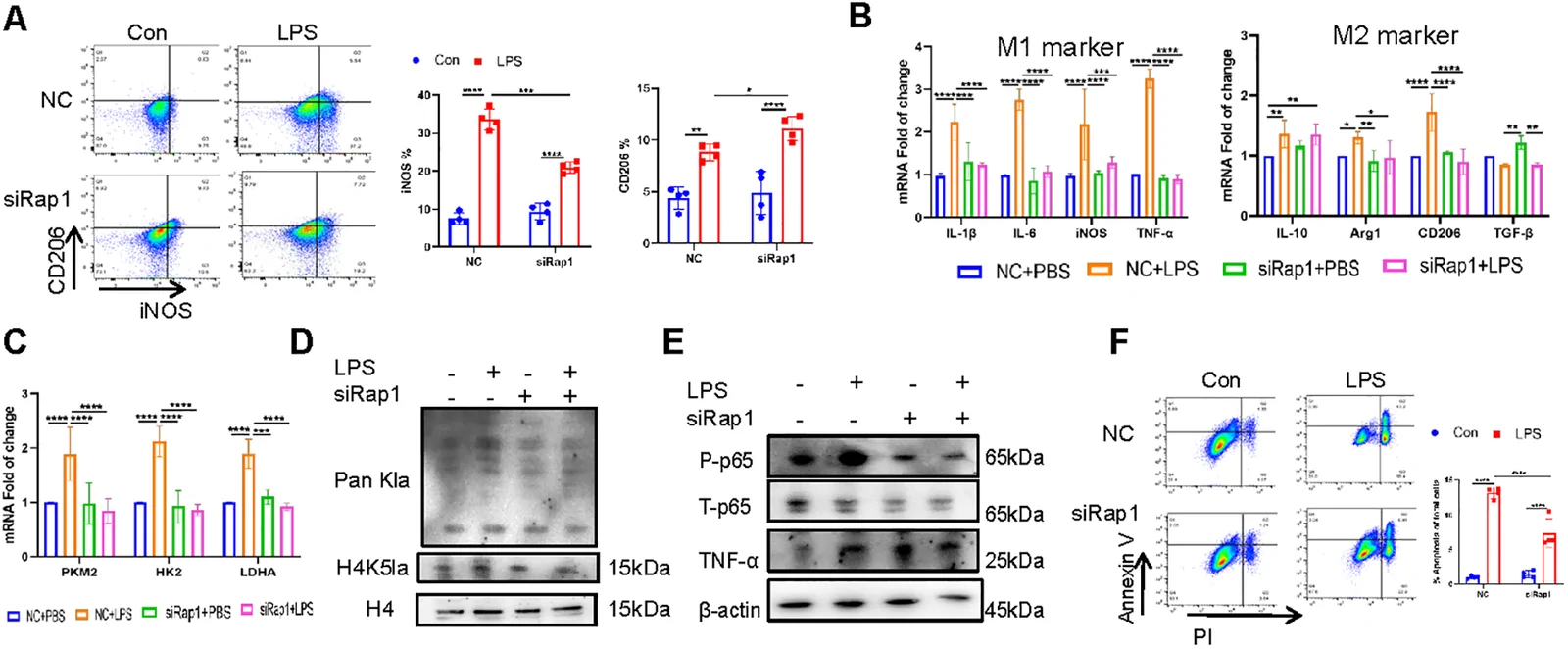
Knockdown of Rap1 reduces macrophage-mediated inflammatory damage. **(A)** Representative flow cytometric and statistical analysis of M1 macrophages and M2 macrophages in the BMDMs treated with LPS and siRap1 (n=4 biological replicates). **(B)** Relative gene expression of M1-macrophage markers and M2-macrophage markers mRNA was normalized to 18S mRNA in the BMDMs treated with LPS and siRap1 (n=4 biological replicates). **(C)** Relative gene expression of glycolysis markers mRNA was normalized to 18S mRNA in the BMDMs treated with LPS and siRap1 (n=4 biological replicates). **(D)** Representative immunoblots of Panla and H4K5la in the BMDMs treated with LPS and siRap1. H4 was shown as the loading control. **(E)** Representative immunoblots of P-p65, T-p65 and TNF-a in the BMDMs treated with LPS and siRap1. β-actin was shown as the loading control. **(F)** Representative flow cytometric and statistical analysis of apoptotic cells in the BMDMs treated with LPS and siRap1 (n=4 biological replicates). Data were analyzed by Two-way ANOVA followed by Bonferroni test. *p <0.05;**p < 0.01; ***p < 0.005 and ****p < 0.001.

Subsequently, we investigated the role of Rap1 in macrophages by siRNA-mediated knockdown *in vitro*. Q-PCR and western blot results confirmed the knockdown efficiency, leading to the selection of siRap1–1 for the subsequent experiments (**Supplementary Figures 5C, D**). The flow cytometry analysis showed that Rap1 knockdown suppressed the pro-inflammatory M1 polarization induced by LPS (**Figure 5A**). Consistently, Q-PCR and western blot analysis indicated that the RNA and protein expression levels of M1-subtype and M2-subtype genes were significantly altered in the siRap1-treated macrophages (**Figure 5B**; **Supplementary Figure 5E**). Importantly, we observed that the expression of glycolysis genes in BMDMs was significantly reduced when treated with the siRap1 (**Figure 5C**; **Supplementary Figure 5F**). The protein levels of Panla and H4K5la were decreased following the Rap1 knockdown (**Figure 5D**). Silencing of Rap1 significantly attenuated the LPS-triggered activation of the TNF/NF-κB signaling axis (**Figure 5E**). Moreover, suppression of Rap1 expression inhibited the inflammatory injury, which was accompanied by a reduction in both apoptosis and oxidative stress (**Figure 5F**; **Supplementary Figure 5G**). Together, these results suggest Rap1 as a key mediator linking inflammation and glycolytic metabolism in macrophages.

### Macrophage-specific Rap1 ablation ameliorates acute myocarditis

**Figure 6:**
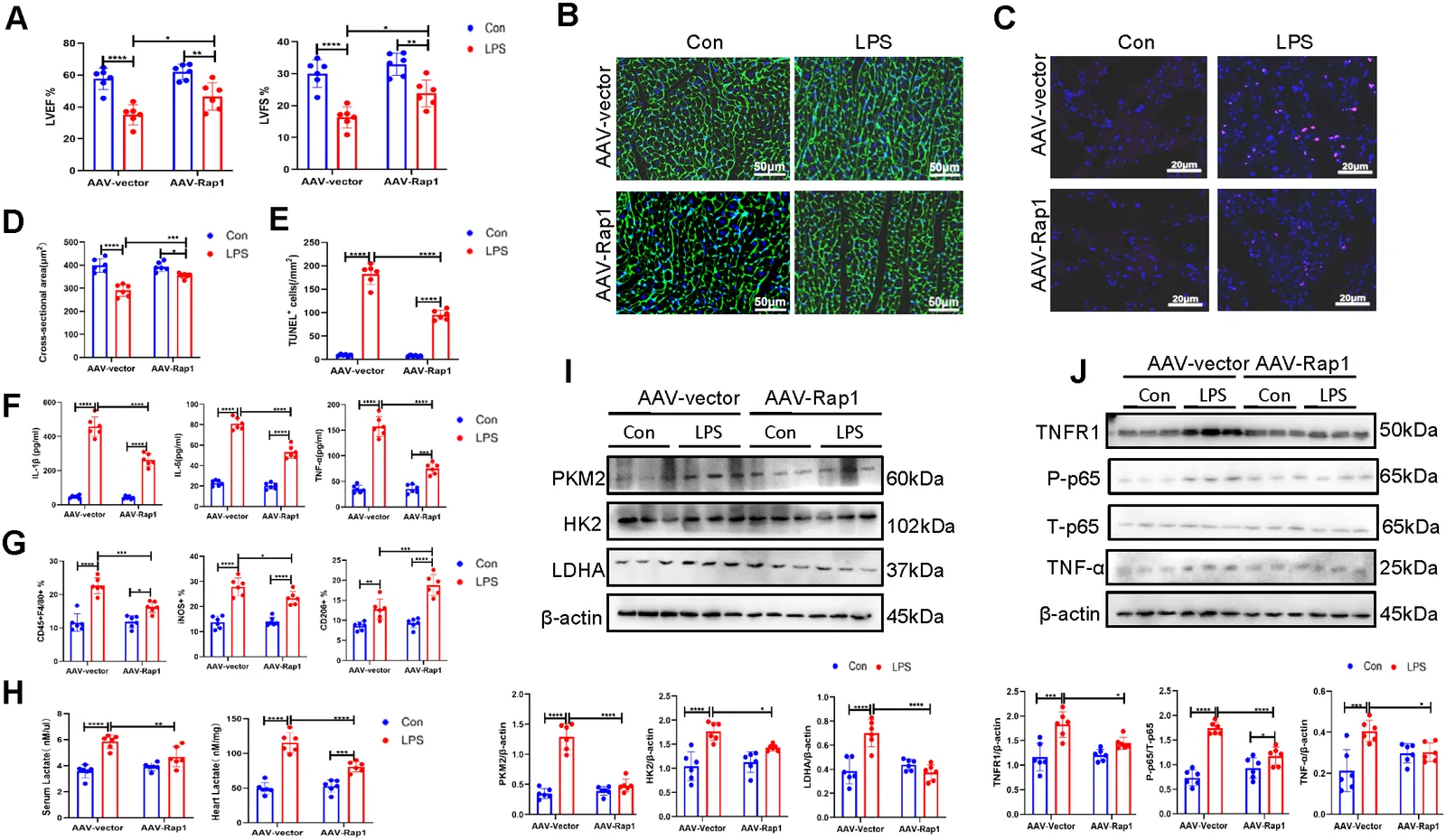
Inhibition of Rap1 in macrophage rescues cardiac function in acute myocarditis. **(A)** Echocardiography measurements of cardiac function of mice. LVEF and LVFS were shown at day 1 after LPS injection (n=6 each group). **(B)** Representative immunofluorescence images of cardiac sections stained with myocyte membrane (WGA, green) and nuclei (DAPI, blue) from mice. Scale bar=50 μm. **(C)** Representative immunofluorescence images of cardiac sections stained with apoptotic cells (TUNEL, pink) and nuclei (DAPI, blue) from mice. Scale bar=20 μm. **(D)** Quantification of cross-sectional cell area identified by WGA staining in B (n=6 each group). **(E)** Quantifications of TUNEL^+^ cells identified by TUNEL staining in C (n=6 each group). **(F)** ELISA analysis of inflammatory factor IL-1, IL-6 and TNF-α in murine plasma (n=6 each group). **(G)** Flow cytometric statistical analysis of total macrophages, M1 macrophages and M2 macrophages in the cardiac tissues (n=6 each group). **(H)** Quantification of lactate content in the heart tissues and serum (n=6 each group). **(I)** Representative immunoblots and the corresponding analysis of glycolysis associated proteins in the cardiac tissues from mice. β-actin was shown as the loading control (n=6 each group). **(J)** Representative immunoblots and the corresponding analysis of TNF and NF-kB pathway proteins in the cardiac tissues from mice (n=6 each group). β-actin was shown as the loading control. Data were presented as mean ± SD. Data were analyzed by Two-way ANOVA followed by Bonferroni test. *p <0.05;**p < 0.01; ***p < 0.005 and ****p < 0.001.

To evaluate the effect of Rap1 on acute myocarditis, we constructed a myeloid-specific CD11b promoter-driven AAV9 encoding *Rap1*. Following 21 days of transfection in mice, the protein levels of Rap1 in the BMDMs as well as the hearts of the mice were examined (**Supplementary Figure 6A**). Suppression of Rap1 expression attenuated LPS-mediated reduction in cardiac function (**Figure 6A**). Moreover, Rap1 knockdown ameliorated the worsening effects of LPS treatment, including cardiomyocyte shrinkage and apoptosis (**Figures 6B–E**). Of note, ablation of Rap1 decreased the expression of inflammatory cytokines both in the circulation and hearts of LPS-treated mice (**Figure 6F**; **Supplementary Figure 6B**). Flow cytometry analyses further indicated that Rap1 inhibition led to a decrease in the mobilization and infiltration of macrophage as well as the pro-inflammatory subtype polarization (**Figure 6G**; **Supplementary Figure 6C**). Next, we observed that the lactate contents of serum and the tissues were reduced in the Rap1 knockdown group compared to the vector group (**Figure 6H**). Consistently, the proteins expression of glycolysis pathway and H4K5la were also attenuated by Rap1 ablation (**Figure 6I**; **Supplementary Figure 6D**). Myeloid-specific depletion of Rap1 resulted in diminished TNF/NF-κB signaling in response to LPS (**Figure 6J**). In addition, we found that knockdown of Rap1 in macrophages rescued the long-term ventricular remodeling and fibrosis at 28 day after LPS injection (**Supplementary Figures 6E, F**). Collectively, these findings suggest that H4K5la-driven upregulation of Rap1 in macrophages exacerbates inflammation through TNF/NF-κB signaling, thereby establishing a feed-forward loop that promotes sustained lactate production and H4K5la during myocarditis.

## Discussion

**Figure 7:**
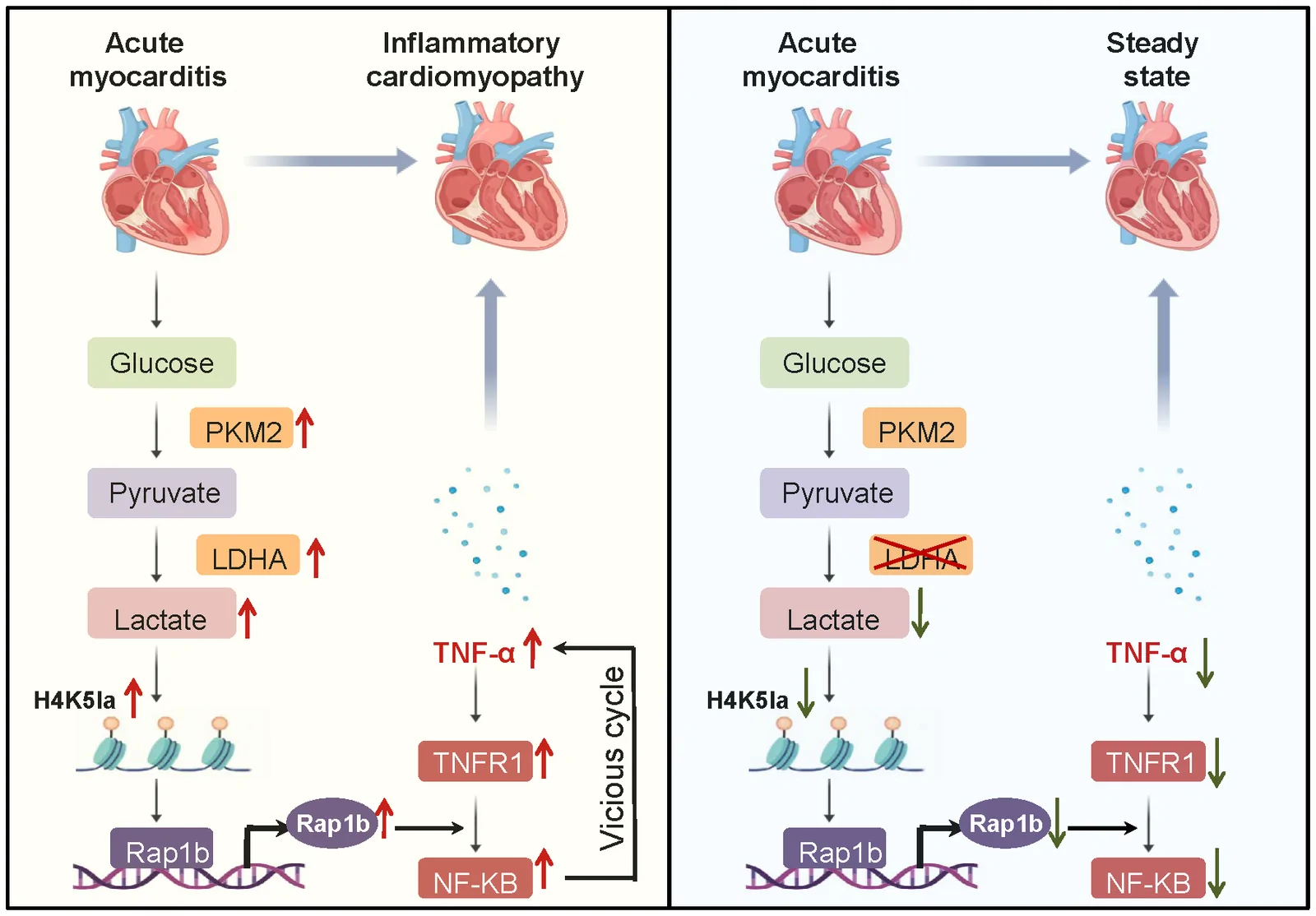
H4K5 lactylation in macrophages exacerbates acute myocarditis via a Rap1/TNF–NF-κB-driven pro-inflammatory feedback loop.

Acute myocarditis undergoes a shift to aerobic glycolysis in response to inflammatory cascades. It has been reported that glycolysis-mediated lactate production and protein lactylation play a pivotal role in orchestrating the inflammatory response (23). In this study, we discovered that acute myocarditis enhances glycolysis in macrophages, resulting in lactate accumulation. This accumulated lactate significantly increases the levels of Panla and H4K5la in macrophages. Notably, H4K5la upregulates the transcription of the inflammatory factor Rap1, which in turn activates the TNF/NF-κB pathway. This cascade exacerbates macrophage activation and dysfunction, ultimately driving inflammatory cardiomyopathy (**Figure 7**). Our findings highlight the role of H4K5la in macrophage and reveal a novel mechanism by which lactylation participates in myocarditis.

Currently, therapeutic options for myocarditis remain limited. The inflammatory response is the core pathological mechanism of myocarditis, featuring a complex network with diverse targets. In the early stage of inflammation, activated innate immune cells trigger inflammatory responses to counteract invading pathogens. However, inadequate management of the initial response may result in exacerbated inflammatory reactions, potentially leading to severe compromise of cardiac function. Metabolism signaling is determinant for cardiac function, and the feature of metabolic reprogramming is increased glycolysis in the progression of heart failure (13). In response to inflammatory stimuli, most of glucose is metabolized to lactate, subsequently generating adenosine triphosphate through aerobic glycolysis, whereas only a minimal fraction is entering into oxidative phosphorylation. These metabolic alterations lead to mitochondrial dysfunction and exacerbating lactate accumulation (24). Lactate was initially regarded as a metabolic waste product, its circulating levels are closely related to the prognosis as well as the severity of the myocarditis (25). Recent research indicates that lactate plays a regulatory role in immune response via post-translational lactylation modification of histone and non-histone proteins (26). Our work demonstrated that lactate accumulation contributes to the progression of acute myocarditis, and inhibition of lactate production could reduce the levels of lactylation and rescued the ventricular remodeling (**Figure 1**).

Macrophages are the center immune cells of the dysregulated inflammatory cascade in myocarditis. At the onset of inflammation, circulating macrophages are recruited to the damaged myocardium and differentiate into activated macrophages. These activated macrophages exhibit functional plasticity by switching between pro-inflammatory and anti-inflammatory phenotypes in response to local inflammatory and metabolic cues. During the acute phase of inflammation, macrophage metabolism shifts toward glycolysis with reduced reliance on oxidative phosphorylation (OXPHOS), leading to elevated lactate levels and polarization toward a pro-inflammatory phenotype. In contrast, during the subsequent immunosuppressive stage, macrophages revert to OXPHOS dependency and upregulate fatty acid oxidation, transitioning to an anti-inflammatory phenotype to facilitate tissue repair (27). Emerging evidences have found that accumulated lactate due to inflammation increases lactylation of macrophages and transcription of specific genes (28). Exogenous lactate administration in LPS-stimulated macrophages drives their transition from a pro-inflammatory phenotype to a tissue-repair phenotype by inducing lactylation of histones [e.g., H3K18la (15)] and non-histone proteins [e.g., PKM2 (29), HMGB1 (30)]. However, the metabolic role of lactate in immune regulation remains debated. Ma et al. found that H4K12la and H3K18la modification on macrophages stabilizes hypoxia-inducible factor-1α (HIF-1α) and promotes the production of inflammatory cytokines such as IL-1β (31). Qiao et al. demonstrated that H3K18la triggers the RhoA/ROCK/Ezrin cascade, which promotes NF-κB activation, increases apoptosis, and exacerbates kidney injury in sepsis (32). Our study shows that inhibiting lactate accumulation in macrophages helps reduce the TNF-NF-κB pro-inflammatory loop and alleviates acute myocarditis. These differences suggest that the effects of lactate may depend on its concentration, the timing of exposure, and the context. On one hand, it serves as an alternative energy source supporting cell survival during tissue injury. On the other hand, excessive lactate accumulation can lead to cytotoxicity and immunometabolic reprogramming. Specifically, histone lactylation increases in a time-dependent manner. Zhang et al. found that M1 macrophages increase lactylation through glycolysis (within 24 hours), and this histone lactylation subsequently activates specific tissue repair genes, driving the transition from M1 to M2 phenotype (15). Bohaud et al. identify the role of lactate at the onset of macrophage differentiation toward a pro-inflammatory phenotype (8). We speculate that excessive lactate accumulation may exceed its utilization capacity in the early stage of inflammation and activate epigenetic programs such as those driven by H4K5la, which regulate genes associated with sustained inflammation (**Figure 2**). Blocking lactate production in macrophages helps alleviate its pro-inflammatory effects (**Figure 3**). Similarly, studies have shown that inhibiting lactylation sites or related signaling pathways can attenuate sepsis pathogenesis and improve patient outcomes (33, 34). Our findings reveal a novel molecular mechanism underlying the pleiotropic effects of lactate.

To date, there are 14 lysine lactylation sites have been identified, distributed across H2A, H2B, H3, and H4 (15). Among these, H3K18la and H4K12la are the most extensively studied. Recent studies have begun to recognize the non-negligible role of H4K5la in regulating inflammation. For example, upregulation of H4K5la in microglia exacerbates inflammatory injury in ischemic stroke (35). A prospective observational study has reported that H4K5la in immune cells is associated with outcomes in patients with sepsis (36). Our study found that H4K5la is upregulated in cardiac macrophages, and blocking lactate production in macrophages significantly reduces H4K5la levels. Further CUT&Tag sequencing revealed that H4K5la target genes are significantly enriched in inflammatory and metabolic signaling such as TNF and NF-κB pathways. Integrated RNA-seq analysis combined with ChIP-PCR validation identified that H4K5la is enriched at the promoter region of Rap1 in macrophages, increasing Rap1 expression and promoting an inflammatory loop (**Figure 4**). These findings reveal the significant pathological relevance of H4K5la in acute myocarditis by regulating macrophage metabolic reprogramming and polarization.

Recent studies have identified P300/CBP as the writer of H4K5la under inflammation conditions (37, 38). Consistent with this, our results also showed that P300 knockdown inhibited the elevation of H4K5la and Rap1, suggesting that H4K5la promotes Rap1 expression in a p300-dependent manner. Lysine residues are subject to various protein modifications, including acetylation, methylation, and ubiquitination. H4K5, as a known acetylation site, defines the stability of BRD4 interactions with chromatin (39). In addition, Li et al. reported that H4K5la and H4K5 acetylation exist in a dynamic competitive state under hypoxic conditions (35). Our results showed that as the duration of inflammatory stimulation increases, the acetylation modification at the H4K5 site is replaced by lactylation modification, indicating that the effect of lactate accumulation plays a significant role in the regulation of the genome.

The TNF/NF-κB pathway is a classic inflammatory signaling cascade. Specifically, when TNF-α binds to TNFR1 on the cell membrane, it induces receptor trimerization and recruitment of adaptor proteins, which in turn activate the IKK complex. IKK then phosphorylates IκB, leading to NF-κB translocation into the nucleus. Subsequently, NF-κB initiates transcription of the TNF-α, thereby synergistically amplifying the inflammatory response (40). Rap1 (Terf2IP), a telomeric repeat-binding factor 2-interacting protein 1, is a newly identified regulator of the NF-κB pathway (41). In addition to its localization at telomeres, Rap1 is ectopically expressed in the cytoplasm, where it forms a complex with IKKs. This complex is essential for recruiting IKKs to the p65 subunit of NF-κB and for enabling its transcriptional activity. Rap1 mutant mice exhibit defective NF-κB activation. Furthermore, Rap1 levels are positively regulated by NF-κB (42). It is reported that elevated cytoplasmic Rap1 levels are observed in human breast cancer cells with hyperactive NF-κB (43). Nevertheless, its role in myocarditis remains underexplored. Our results demonstrate for the first time that H4K5la initiates the transcriptional activity of Rap1, and reveal that Rap1 mediates the TNF/NF-κB cascade to amplify inflammatory injury (**Figure 5**). Our findings confirm that inhibiting Rap1 expression in macrophages effectively reduces inflammation and attenuates ventricular remodeling and the progression of cardiomyopathy induced by acute myocarditis (**Figure 6**).

Although our primary model was LPS-induced myocarditis, we also employed the α-MyHC-induced EAM model to further validate the role of lactate. In this model, we confirmed the accumulation of lactate in the inflamed myocardium and demonstrated that inhibiting lactate production ameliorated EAM-induced cardiac dysfunction. Furthermore, a previous prospective observational study reported that H4K5la levels in immune cells are associated with clinical outcomes in patients with sepsis (36). Given that up to 60% of patients with severe sepsis develop acute myocarditis (44), we speculate that H4K5la and its downstream pathway may have prognostic relevance in myocarditis, although clinical validation is still required. In addition, subsequent studies incorporating both sexes are needed to eliminate potential sex bias.

In summary, our study demonstrates that acute myocarditis triggers a dramatic elevation of glycolysis, leading to intracellular lactate accumulation and subsequent H4K5la modification in macrophages. H4K5la initiates the transcription of Rap1, promoting sustained activation of the TNF/NF-κB signaling and forming a positive feedback loop that amplifies the inflammatory cascade, ultimately driving the progression of inflammatory cardiomyopathy. This network links energy metabolism, epigenetic, and cardiac inflammation. Our findings suggest that H4K5la may serve as a target for acute myocarditis, and that inhibiting lactate production could represent an effective therapeutic strategy for the treatment of myocarditis.

## Data Availability

The datasets presented in this study can be found in online repositories. The names of the repository/repositories and accession number(s) can be found below: PRJNA1474196 (SRA).

## References

[1] Ammirati E Moslehi JJ . Diagnosis and treatment of acute myocarditis: a review. Jama. (2023) 329:1098–113. doi: 10.1001/jama.2023.3371 37014337

[2] Tschope C Ammirati E Bozkurt B Caforio ALP Cooper LT Felix SB . Myocarditis and inflammatory cardiomyopathy: current evidence and future directions. Nat Rev Cardiol. (2021) 18:169–93. doi: 10.1038/s41569-020-00435-x 33046850 PMC7548534

[3] Sagar S Liu PP Cooper LT Jr . Myocarditis. Lancet. (2012) 379:738–47. doi: 10.1016/S0140-6736(11)60648-X 22185868 PMC5814111

[4] Hua X Hu G Hu Q Chang Y Hu Y Gao L . Single-cell RNA sequencing to dissect the immunological network of autoimmune myocarditis. Circulation. (2020) 142:384–400. doi: 10.1161/CIRCULATIONAHA.119.043545 32431172

[5] Huang CH Vallejo JG Kollias G Mann DL . Role of the innate immune system in acute viral myocarditis. Basic Res Cardiol. (2009) 104:228–37. doi: 10.1007/s00395-008-0765-5 19159057 PMC4541794

[6] Liu Y Xu R Gu H Zhang E Qu J Cao W . Metabolic reprogramming in macrophage responses. biomark Res. (2021) 9:1. doi: 10.1186/s40364-020-00251-y 33407885 PMC7786975

[7] Andersson AK Ronnback L Hansson E . Lactate induces tumour necrosis factor-alpha, interleukin-6 and interleukin-1beta release in microglial- and astroglial-enriched primary cultures. J Neurochem. (2005) 93:1327–33. doi: 10.1111/j.1471-4159.2005.03132.x 15934951

[8] Bohaud C Cruz J Terraza C Barthelaix A Laplace-Builhe B Jorgensen C . Lactate metabolism coordinates macrophage response and regeneration in zebrafish. Theranostics. (2022) 12:3995–4009. doi: 10.7150/thno.65235 35664055 PMC9131269

[9] Lu Y Osis G Zmijewska AA Traylor A Thukral S Wilson L . Macrophage-specific lactate dehydrogenase expression modulates inflammatory function *in vitro*. Kidney360. (2025) 6:197–207. doi: 10.34067/KID.0000000630 39531318 PMC11882262

[10] Williams JW Giannarelli C Rahman A Randolph GJ Kovacic JC . Macrophage biology, classification, and phenotype in cardiovascular disease: JACC macrophage in CVD series (part 1). J Am Coll Cardiol. (2018) 72:2166–80. doi: 10.1016/j.jacc.2018.08.2148 30360826 PMC6209330

[11] Viola A Munari F Sanchez-Rodriguez R Scolaro T Castegna A . The metabolic signature of macrophage responses. Front Immunol. (2019) 10:1462. doi: 10.3389/fimmu.2019.01462 31333642 PMC6618143

[12] Eming SA Murray PJ Pearce EJ . Metabolic orchestration of the wound healing response. Cell Metab. (2021) 33:1726–43. doi: 10.1016/j.cmet.2021.07.017 34384520

[13] Lopaschuk GD Karwi QG Tian R Wende AR Abel ED . Cardiac energy metabolism in heart failure. Circ Res. (2021) 128:1487–513. doi: 10.1161/CIRCRESAHA.121.318241 33983836 PMC8136750

[14] Bao C Ma Q Ying X Wang F Hou Y Wang D . Histone lactylation in macrophage biology and disease: from plasticity regulation to therapeutic implications. Ebiomedicine. (2025) 111:105502. doi: 10.1016/j.ebiom.2024.105502 39662177 PMC11697715

[15] Zhang D Tang Z Huang H Zhou G Cui C Weng Y . Metabolic regulation of gene expression by histone lactylation. Nature. (2019) 574:575–80. doi: 10.1038/s41586-019-1678-1 31645732 PMC6818755

[16] Pan RY He L Zhang J Liu X Liao Y Gao J . Positive feedback regulation of microglial glucose metabolism by histone H4 lysine 12 lactylation in Alzheimer's disease. Cell Metab. (2022) 34:634–48:e6. doi: 10.1016/j.cmet.2022.02.013 35303422

[17] Sheng X Lin H Cole PA Zhao Y . Biochemistry and regulation of histone lysine L-lactylation. Nat Rev Mol Cell Biol. (2026) 27:95–109. doi: 10.1038/s41580-025-00876-7 40830268 PMC12920031

[18] Zhang H Xu A Sun X Yang Y Zhang L Bai H . Self-maintenance of cardiac resident reparative macrophages attenuates doxorubicin-induced cardiomyopathy through the SR-A1-c-Myc axis. Circ Res. (2020) 127:610–27. doi: 10.1161/CIRCRESAHA.119.316428 32466726

[19] Wang W Chu Y Lu Y Xu J Zhao W Liang Z . Skatole alleviates osteoarthritis by reprogramming macrophage polarization and protecting chondrocytes. Res (Wash D C). (2025) 8:604. doi: 10.34133/research.0604 39902346 PMC11788598

[20] Sun X Feng Y Zhao Y Cai Y Cao Z Zhang Y . Maresin 1 ameliorates myocardial ischemia–reperfusion injury by promoting tissue resident macrophage efferocytosis. Cardiovasc Res. (2026). doi: 10.1093/cvr/cvag017 41554295

[21] Wu K Fan D Zhao H Liu Z Hou Z Tao W . Dynamics of histone acetylation during human early embryogenesis. Cell Discov. (2023) 9:29. doi: 10.1038/s41421-022-00514-y 36914622 PMC10011383

[22] Liu P Zhang J Zhang J Yuan Y Liu Z Chen S . Quantitation of global histone post-translational modifications reveal anti-inflammatory epigenetic mechanisms of liquiritigenin based on the optimized super-SILAC strategy. Front Cell Dev Biol. (2025) 13:1566567. doi: 10.3389/fcell.2025.1566567 40213392 PMC11982745

[23] Li X Yang Y Zhang B Lin X Fu X An Y . Lactate metabolism in human health and disease. Signal Transduct Target Ther. (2022) 7:305. doi: 10.1038/s41392-022-01151-3 36050306 PMC9434547

[24] Sun Q Karwi QG Wong N Lopaschuk GD . Advances in myocardial energy metabolism: metabolic remodelling in heart failure and beyond. Cardiovasc Res. (2024) 120:1996–2016. doi: 10.1093/cvr/cvae231 39453987 PMC11646102

[25] Greene SJ Hernandez AF Dunning A Ambrosy AP Armstrong PW Butler J . Hospitalization for recently diagnosed versus worsening chronic heart failure: from the ASCEND-HF trial. J Am Coll Cardiol. (2017) 69:3029–39. doi: 10.1016/j.jacc.2017.04.043 28641792

[26] Zhang J Wu D Zeng F Gu H Li C Cata JP . Lactate metabolic reprogramming and histone lactylation modification in sepsis. Int J Biol Sci. (2025) 21:5034–55. doi: 10.7150/ijbs.116088 40860191 PMC12374839

[27] Trujillo MN Jennings EQ Hoffman EA Zhang H Phoebe AM Mastin GE . Lactoylglutathione promotes inflammatory signaling in macrophages through histone lactoylation. Mol Metab. (2024) 81:101888. doi: 10.1016/j.molmet.2024.101888 38307385 PMC10869261

[28] Xu B Liu Y Li N Geng Q . Lactate and lactylation in macrophage metabolic reprogramming: current progress and outstanding issues. Front Immunol. (2024) 15:1395786. doi: 10.3389/fimmu.2024.1395786 38835758 PMC11148263

[29] Wang J Yang P Yu T Gao M Liu D Zhang J . Lactylation of PKM2 suppresses inflammatory metabolic adaptation in pro-inflammatory macrophages. Int J Biol Sci. (2022) 18:6210–25. doi: 10.7150/ijbs.75434 36439872 PMC9682528

[30] Yang K Fan M Wang X Xu J Wang Y Tu F . Lactate promotes macrophage HMGB1 lactylation, acetylation, and exosomal release in polymicrobial sepsis. Cell Death Differ. (2022) 29:133–46. doi: 10.1038/s41418-021-00841-9 34363018 PMC8738735

[31] Ma XM Geng K Wang P Jiang Z Law BY Xu Y . MCT4-dependent lactate transport: a novel mechanism for cardiac energy metabolism injury and inflammation in type 2 diabetes mellitus. Cardiovasc Diabetol. (2024) 23:96. doi: 10.1186/s12933-024-02178-2 38486199 PMC10941417

[32] Qiao J Tan Y Liu H Yang B Zhang Q Liu Q . Histone H3K18 and Ezrin lactylation promote renal dysfunction in sepsis-associated acute kidney injury. Adv Sci (Weinh). (2024) 11:e2307216. doi: 10.1002/advs.202307216 38767134 PMC11267308

[33] Lu Z Fang P Li S Xia D Zhang J Wu X . Lactylation of histone H3k18 and Egr1 promotes endothelial glycocalyx degradation in sepsis-induced acute lung injury. Adv Sci (Weinh). (2025) 12:e2407064. doi: 10.1002/advs.202407064 39721014 PMC11831459

[34] Liao ST Han C Xu DQ Fu XW Wang JS Kong LY . 4-Octyl itaconate inhibits aerobic glycolysis by targeting GAPDH to exert anti-inflammatory effects. Nat Commun. (2019) 10:5091. doi: 10.1038/s41467-019-13078-5 31704924 PMC6841710

[35] Li Y Zhou Y Mo Y Li Y Wang P Zhao Y . Histone H4 lysine 5 lactylation: a key regulator of immune metabolism in microglia during ischemic stroke. Pharmacol Res. (2026) 225:108127. doi: 10.1016/j.phrs.2026.108127 41638595

[36] Guo Y Chu L Shui W Hu H Hao L Wang D . Histone lactylation in immune cells and its predictive role in sepsis progression: a prospective observational study. Shock. (2025). doi: 10.1097/SHK.0000000000002659 40663442 PMC13132054

[37] Yamane M Akiyama Y Shimada S Watanabe S Hatano M Tsukihara S . H4K5 lactylation - ENO2 loop drives glycolysis and HCC progression. Jhep Rep. (2026) 8:101786. doi: 10.1016/j.jhepr.2026.101786 41962496 PMC13091297

[38] Tang S Liu Y Jin B Zhang T Wang X Zhao J . LDHA facilitates immune escape in bladder cancer through H4K5 lactylation-dependent upregulation of PD-L1. Biochem Pharmacol. (2026) 243:117553. doi: 10.1016/j.bcp.2025.117553 41274423

[39] Gao M Wang J Rousseaux S Tan M Pan L Peng L . Metabolically controlled histone H4K5 acylation/acetylation ratio drives BRD4 genomic distribution. Cell Rep. (2021) 36:109460. doi: 10.1016/j.celrep.2021.109460 34320364

[40] Brenner D Blaser H Mak TW . Regulation of tumour necrosis factor signalling: live or let die. Nat Rev Immunol. (2015) 15:362–74. doi: 10.1038/nri3834 26008591

[41] Teo H Ghosh S Luesch H Ghosh A Wong ET Malik N . Telomere-independent Rap1 is an IKK adaptor and regulates NF-kappaB-dependent gene expression. Nat Cell Biol. (2010) 12:758–67. doi: 10.1038/ncb2080 20622870

[42] Zhang Z Zhou M Tang Y Qi J Xu X Wang P . Impaired megakaryopoiesis due to aberrant macrophage polarization via BTK/Rap1/NF-kappaB pathway in sepsis-induced thrombocytopenia. Mol Ther. (2025) 33:1769–84. doi: 10.1016/j.ymthe.2024.12.048 39741411 PMC11997490

[43] Basak S Hoffmann A . Crosstalk via the NF-kappaB signaling system. Cytokine Growth Factor Rev. (2008) 19:187–97. doi: 10.1016/j.cytogfr.2008.04.005 18515173 PMC2675004

[44] Aissaoui N Boissier F Chew M Singer M Vignon P . Sepsis-induced cardiomyopathy. Eur Heart J. (2025) 46:3339–53. doi: 10.1093/eurheartj/ehaf340 40439150

