# Supplementary material for: H4K5 lactylation exacerbates acute myocarditis by driving a positive feedback loop between inflammation and metabolic dysfunction
Source: Front Immunol. 2026 Aug 24;17:1896701. doi: 10.3389/fimmu.2026.1896701 (PMC13547024; doi:10.3389/fimmu.2026.1896701)
Supplement: Supplementary file 1 [file DataSheet1.pdf]

frontiers

The International Society for the Advancement of Cytometry (ISAC) has highlighted the importance of including comprehensive methodological information to ensure data reproducibility and reliability. In line with this, Frontiers in Immunology now requires authors to submit a checklist for manuscripts that involve flow or mass cytometry. This checklist helps standardize the reporting process, improving the quality and transparency of published data. By doing so, we support scientific progress, making it easier for other researchers to replicate and validate experiments.

This form should be submitted with any manuscripts using flow or mass cytometry.

Sample/specimen/material description

- [x] Total blood
- [ ] PBMCs
- [ ] Organ digests

Other heart tissues and bone marrow-derived macrophages

Did the samples suffer any treatment before or after incubation with the antibodies?

- [x] Drug LPS;FX-11
- [x] Cell permeabilization Fixation/Permeabilization
- [x] Dye DCFDA-FITC fluorescent prob
- [x] Propidium iodine
- [ ] Not applicable

Other Red blood cell lysis

Instrument and antibodies

Name of the Cytometer Beckman Cytoflex S

Antibodies and targets	Fluorochrome/ Metal	Catalog number/Company
e.g. anti-CD4	FITC	Cat. XXX/ XXX ltd.
anti-CD45	APC-Cy7	Cat.557659/BD Biosciences

anti-FVS	eFlour660	Cat.65-0864-14/eBioscie
anti-CD11b	PerCP-Cyanine5.5	Cat.45-0112-82/eBioscie
anti-F4/80	FITC	Cat.123107/Biolgend
anti-iNOS	PE	Cat.696805/Biolgend
anti-CD206	APC	Cat.141707/Biolgend
anti-Annexin V/PI	FITC/PI	Cat.A211-01/Vazyme

Data analyses

1. Name of the software FlowJo_V10_________________________________
2. Reference gating strategy in the manuscript or supplementary material

Gating strategy in (eg Figure X) Figure S2A_______________________________
