# Supplementary material for: H4K5 lactylation exacerbates acute myocarditis by driving a positive feedback loop between inflammation and metabolic dysfunction
Source: Front Immunol. 2026 Aug 24;17:1896701. doi: 10.3389/fimmu.2026.1896701 (PMC13547024; doi:10.3389/fimmu.2026.1896701)
Supplement: Supplementary file 2 [file DataSheet2.pdf]

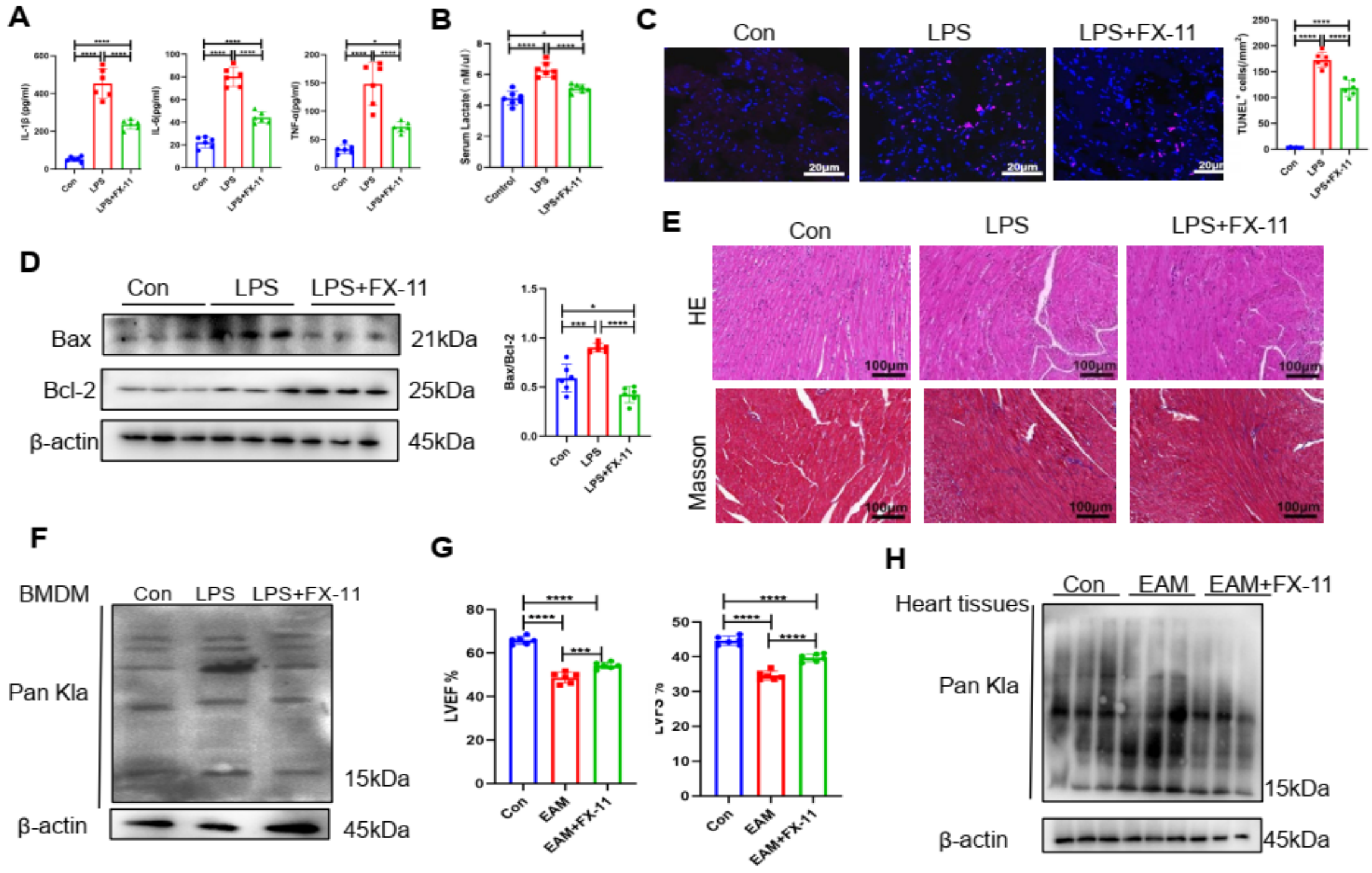

Figure S1. The suppression of lactate production mitigates cardiac damage in acute myocarditis.

A. ELISA analysis of inflammatory factor IL-1, IL-6 and TNF-α in murine plasma (n=10 each group). B. Quantification of lactate content in the serum (n=6 each group). C. Representative immunofluorescence images of cardiac tissues staining with apoptotic cells (TUNEL, pink) and nuclei (DAPI, blue). 5 images per mouse are evaluated. Quantifications of TUNEL+ intensity were shown on right (n=6 each group).Scale bar=20 μm. D. Representative immunoblots and the corresponding analysis of apoptosis associated proteins in the cardiac tissues from mice. β-actin was shown as the loading control (n=6 each group). E. Representative images of HE and Masson staining of heart tissues from mice at 14 day after LPS injection. Scale bar=100 μm. F. Representative immunoblots of Panla protein in the BMDMs from each group. β-actin was shown as the loading control. G. LVEF and LVFS was assessed by echocardiography at 21 day after EAM (n=6 each group). H. Representative immunoblots of Panla protein in the cardiac tissues from each group. β-actin was shown as the loading control. Data were presented as mean±SD. Data were analyzed by One-way ANOVA followed by Bonferroni test. *p <0.05;**p < 0.01; ***p < 0.005 and ****p < 0.001.

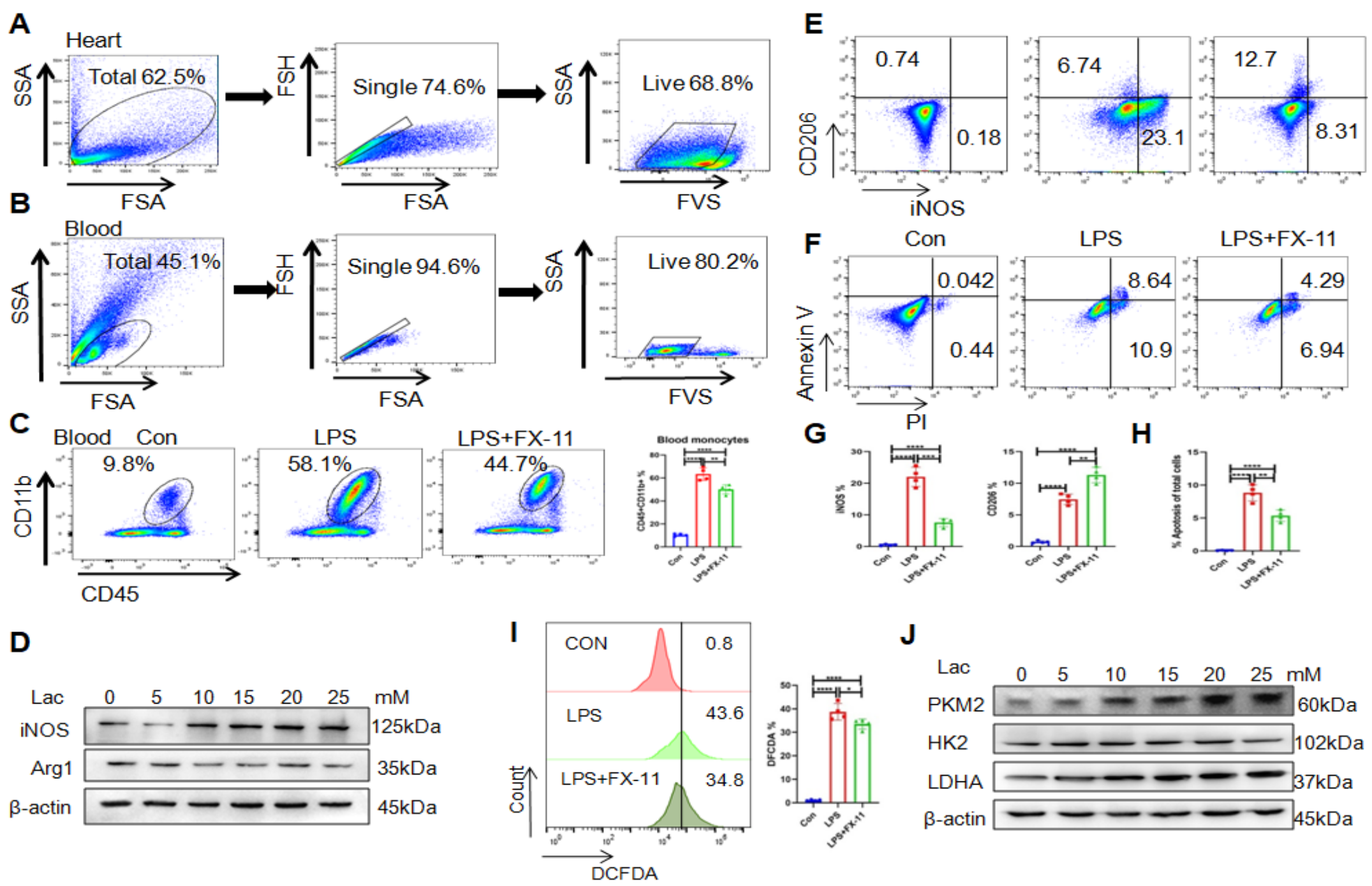

Figure S2. Lactate induces pro-inflammatory M1 polarization in macrophages. A-B. Flow cytometry gating strategy utilized to identify and characterize live cells populations in the hearts and blood of mice. C. Representative flow cytometric and statistical analysis of total monocytes in the blood of mice (n=4 each group). D. Representative immunoblots and the corresponding analysis of M1 macrophage marker iNOS and M2 macrophage marker Arg1 proteins in the BMDMs treated with different concentrations of lactate. β-actin was shown as the loading control. E. Representative flow cytometric of $iNOS^+$M1/$CD206^+$M2 subtype macrophages in the BMDMs treated with LPS and FX-11. F. Representative flow cytometric of apoptotic BMDMs treated with LPS and FX-11. G. Statistical analysis corresponding to the $iNOS^+$M1/$CD206^+$M2 subtype macrophages in the E (n=4 biological replicates). H. Statistical analysis corresponding to the apoptotic macrophages in the F (n=4 biological replicates). I. Representative flow cytometric and statistical analysis of DCFDA intensity in the BMDMs treated with LPS and FX-11 (n=4 biological replicates). J. Representative immunoblots and the corresponding analysis of glycolysis associated proteins in the BMDMs treated with different concentrations of lactate. β-actin was shown as the loading control. Data were presented as mean±SD. Data were analyzed by One-way ANOVA followed by Bonferroni test. *p <0.05;**p < 0.01; ***p < 0.005 and ****p < 0.001.

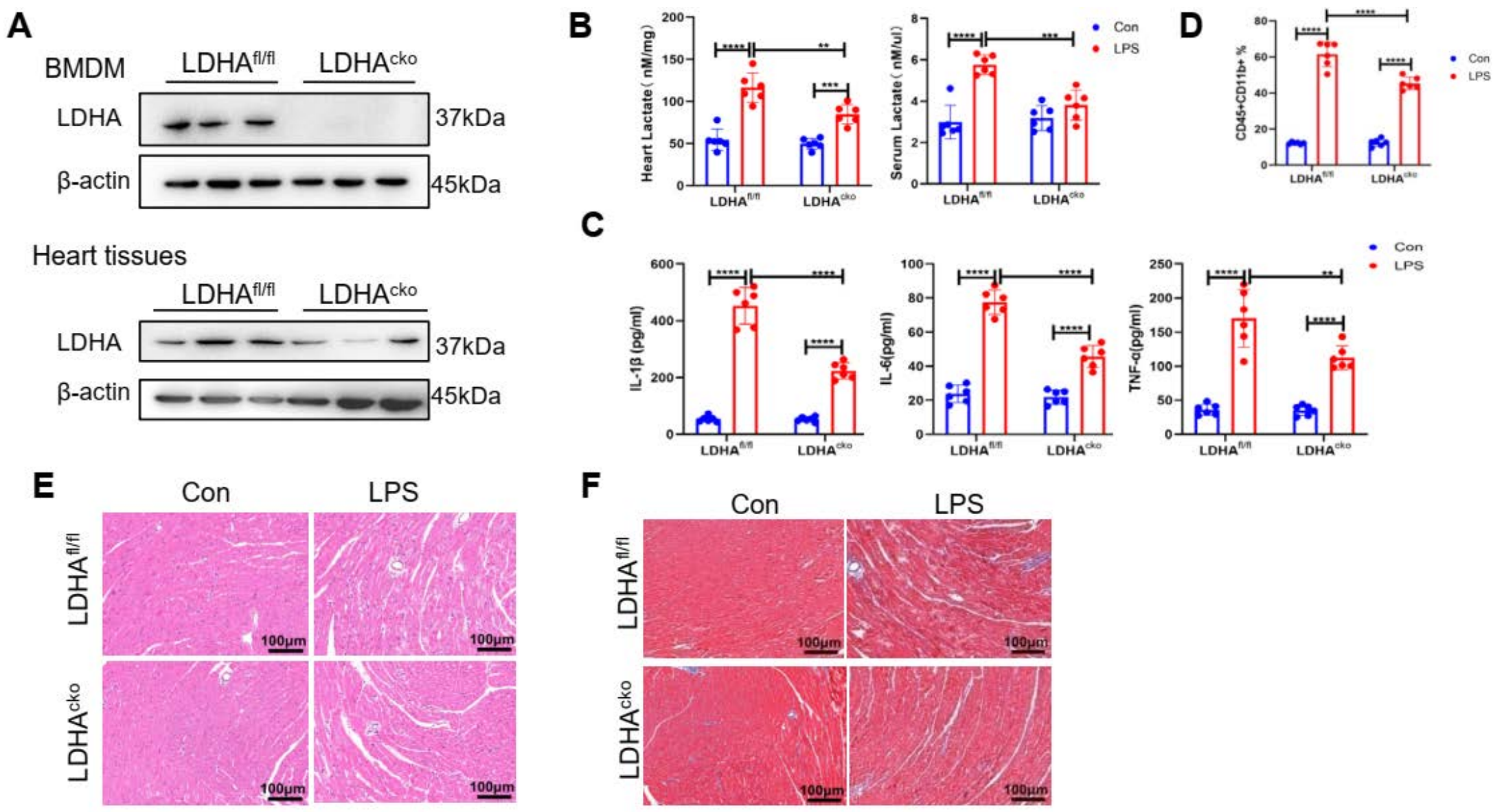

Figure S3. Myocarditis injury can be mitigated by suppressing lactate production in macrophages.

A. Representative immunoblots of LDHA proteins in the BMDMs and cardiac tissues from LDHA$^{fl/fl}$ and LDHA^{cko} mice. β-actin was shown as the loading control. B. Quantification of lactate content in the heart tissues and serum from LDHA$^{fl/fl}$ and LDHA^{cko} mice (n=6 each group). C. The serum levels of proinflammatory cytokines IL-1β, IL-6 and TNF-α were measured in the LDHA$^{fl/fl}$ and LDHA^{cko} mice treated with or without LPS (n=6 each group). D. Flow cytometry analysis of CD45^{+}CD11b^{+} total monocytes in the circulation from LDHA$^{fl/fl}$ and LDHA^{cko} mice (n=6 each group). E-F. Representative images of HE and Masson staining of cardiac sections from LDHA$^{fl/fl}$ and LDHA^{cko} mice at 14 day after LPS injection. Scale bar=100 μm. Data were presented as mean±SD. Data were analyzed by Two-way ANOVA followed by Bonferroni test. *p <0.05;**p < 0.01; ***p < 0.005 and ****p < 0.001.

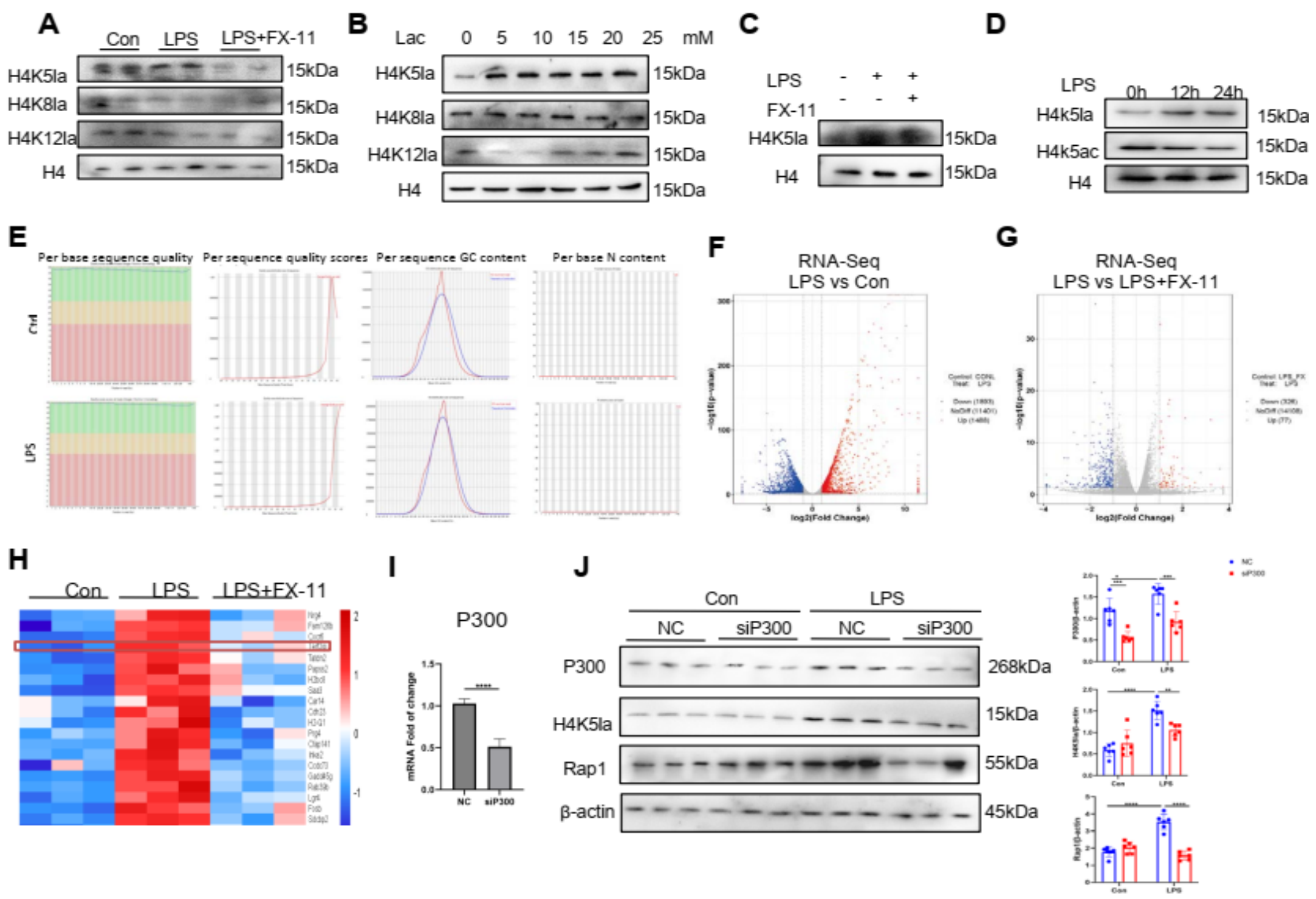

Figure S4. H4K5 lactylation was increased in the lactate-treated macrophages.

A. Representative immunoblots of H4K5la, H4K8la and H4K12la in the cardiac tissues from each group were shown. H4 was shown as the loading control. B. Representative immunoblots of H4K5la, H4K8la and H4K12la in the BMDMs treated with Lac (0, 5, 10, 15, 20, 25 mM). H4 was shown as the loading control. C. Representative immunoblots of H4K5la protein in the BMDMs treated with LPS and FX-11. H4 was shown as the loading control. D. Representative immunoblots of H4K5la and H4K5ac in the BMDMs treated with LPS after different time point. H4 was shown as the loading control. E. The quality metrics for the CUT&Tag sequencing data. F. Volcano plot from RNA sequencing profiling in BMDMs (LPS vs Con). G. Volcano plot from RNA sequencing profiling in BMDMs (LPS vs LPS+FX-11). H. Heatmap produced from the RNA sequencing in BMDMs. I.Relative gene expression of P300 mRNA was normalized to 18S mRNA in the BMDMs treated with siP300 (n=4 biological replicates). J. Representative immunoblots of P300, H4K5la and Rap1 proteins in the BMDMs treated with LPS and siP300. β-actin was shown as the loading control (n=6 biological replicates). Data of I was analyzed by Student t-test; Data of J were analyzed by Two-way ANOVA followed by Bonferroni test. *p <0.05;**p < 0.01; **p < 0.01 and ****p < 0.001

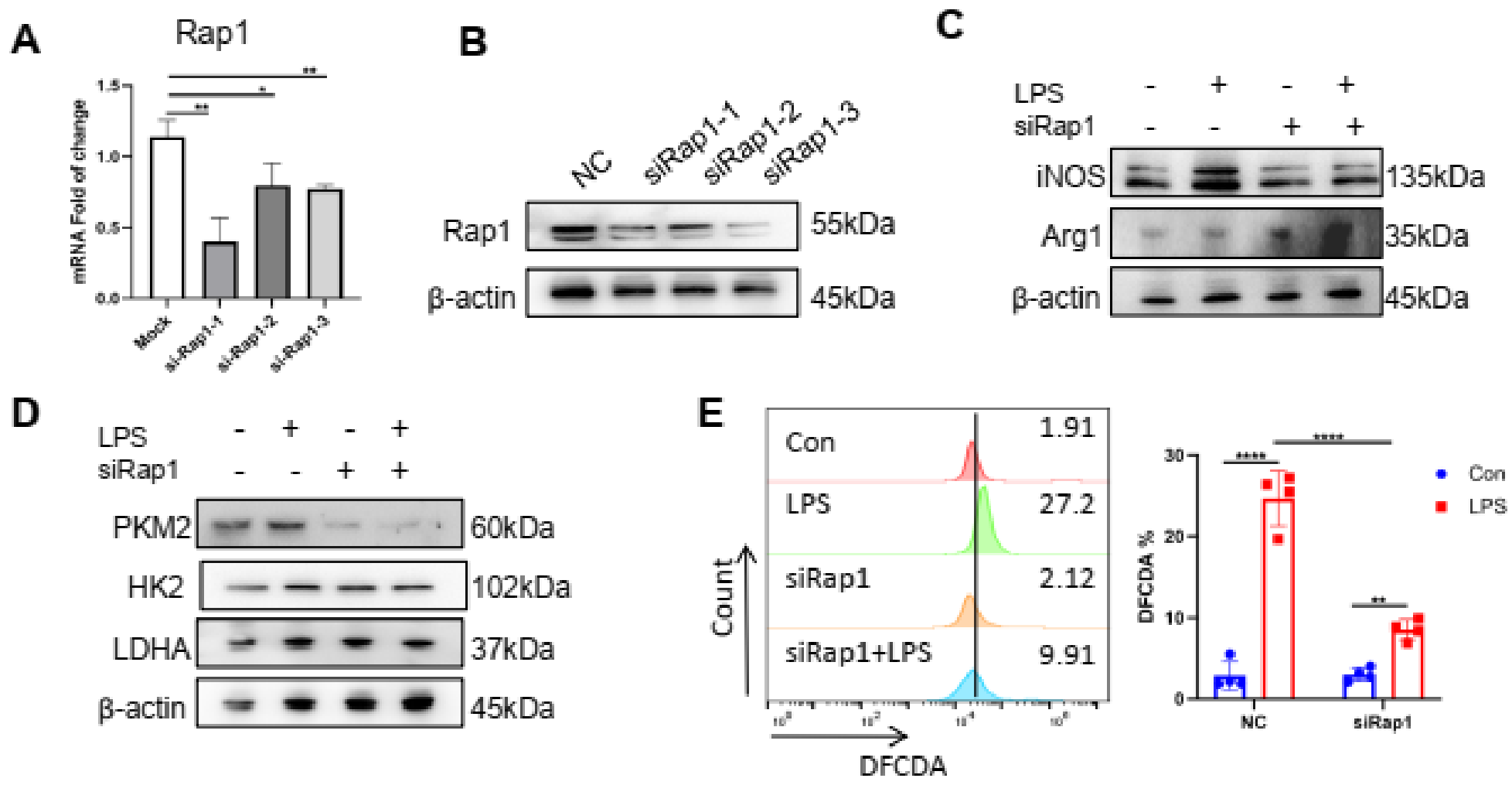

Figure S5. Inhibition of Rap1 in vitro reduced the polarization of macrophages towards a pro-inflammatory phenotype.

A. C. Relative gene expression of Rap1 mRNA was normalized to 18S mRNA in the BMDMs treated with siRap1 (n=4 biological replicates). D. Immunoblots analysis of Rap1 in the BMDMs treated with siRap1. β-actin was shown as the loading control. E. Representative immunoblots of M1 macrophage marker iNOS and M2 macrophage marker Arg1 proteins in the BMDMs treated with LPS and siRap1. β-actin was shown as the loading control. F. Representative immunoblots of glycolysis associated proteins in the BMDMs treated with LPS and siRap1. β-actin was shown as the loading control. G. Representative flow cytometric and statistical analysis of DCFDA intensity in the BMDMs treated with LPS and siRap1 (n=4 biological replicates). Data of C were analyzed by One-way ANOVA followed by Bonferroni test; Data of E were analyzed by Two-way ANOVA followed by Bonferroni test;. *p <0.05;**p < 0.01; **p < 0.01 and ****p < 0.001.

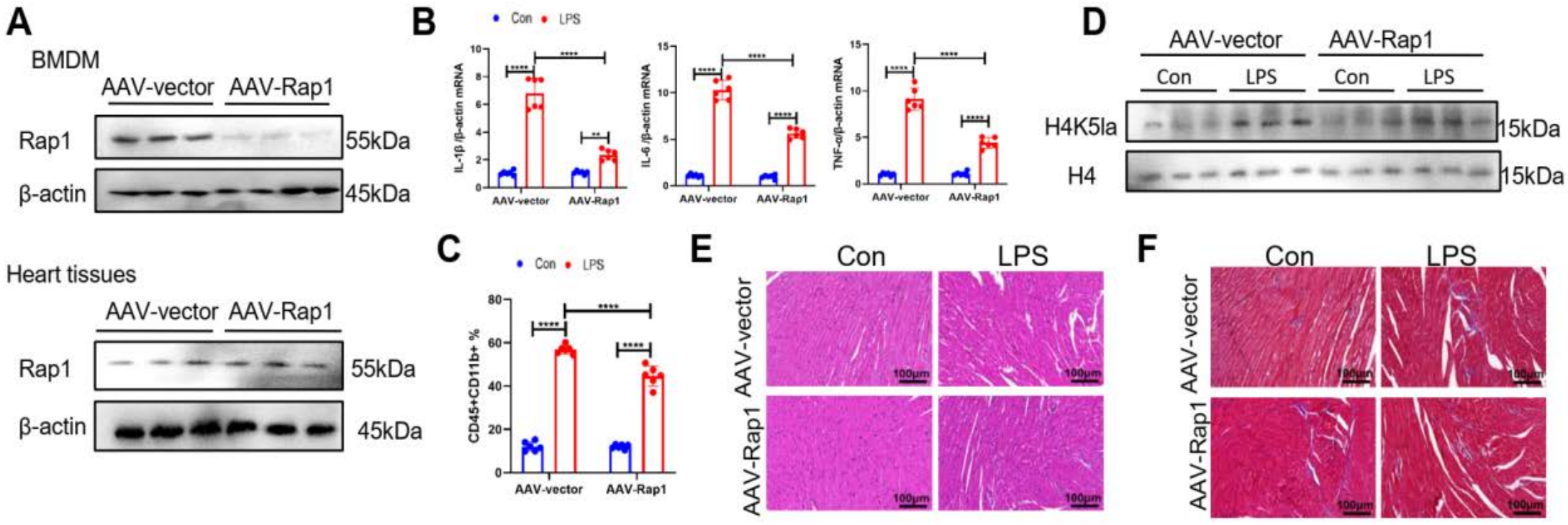

Figure S6. Targeted ablation of Rap1 in macrophages attenuates acute myocarditis.

A. Representative immunoblots and the corresponding analysis of Rap1 proteins in the BMDMs and cardiac tissues from mice transfected with AAV-vector or AAV-Rap1. β-actin was shown as the loading control. B. The mRNA levels of inflammatory cytokines IL-1β, IL-6 and TNF-α were measured in the cardiac tissues from mice (n=6 each group). C. Flow cytometry analysis of $CD45^{+}CD11b^{+}$ total monocytes in the circulation from mice (n=6 each group). D. Representative immunoblots of H4K5la in the cardiac tissues from mice. H4 was shown as the loading control. E. Representative images of HE staining of cardiac sections from mice at 14 day after LPS injection. Scale bar=100 μm. F. Representative images of Masson staining of cardiac sections from mice at 14 day after LPS injection. Scale bar=100 μm. Data were presented as mean±SD. Data were analyzed by Two-way ANOVA followed by Bonferroni test. *p <0.05;**p < 0.01; ***p < 0.005 and ****p < 0.001.
